# Effectiveness of Vitamin D Supplementation on Biochemical, Clinical, and Inflammatory Parameters in Patients with Different Types of Diabetes: A Systematic Review and Meta-Analysis

**DOI:** 10.3390/nu17182991

**Published:** 2025-09-18

**Authors:** Alejandro Bruna-Mejías, Rocío Valdivia-Arroyo, Emelyn Sofia Becerra-Rodríguez, Ignacio Clasing-Cárdenas, Yesica Tatiana Castaño-Gallego, Guinevere Granite, Mathias Orellana-Donoso, Gustavo Oyanedel-Amaro, Pablo Nova-Baeza, Gloria Cifuentes-Suazo, Alejandra Suazo-Santibañez, Juan Sanchis-Gimeno, Héctor Gutiérrez Espinoza, Juan José Valenzuela-Fuenzalida

**Affiliations:** 1 Departamento de Ciencias y Geografía, Facultad de Ciencias Naturales y Exactas, Universidad de Playa Ancha, Valparaíso 2360072, Chile; alejandro.bruna@upla.cl; 2 Department of Morphology, Faculty of Medicine, Andrés Bello University, Santiago 8370146, Chile; rovaldiviaa04@gmail.com (R.V.-A.); emelyn.becerra01@uceva.edu.co (E.S.B.-R.); ignacio.clasing@gmail.com (I.C.-C.); yesica.castano01@uceva.edu.co (Y.T.C.-G.); pablo.nova@gmail.com (P.N.-B.); 3 Department of Surgery, Uniformed Services University of the Health Sciences, Bethesda, MD 20817, USA; guinevere.granite@usuhs.edu; 4 Escuela de Medicina, Universidad Finis Terrae, Santiago 7501015, Chile; mathor94@gmail.com; 5 Faculty of Medicine and Science, Universidad San Sebastián, Santiago 8420524, Chile; 6 Facultad de Ciencias de la Salud, Universidad Autónoma de Chile, Santiago 8910060, Chile; g.oyanedelamaro@gmail.com; 7 Facultad de Medicina, Carrera de Odontología, Universidad Católica de la Santísima Concepción, Av. Alonso de Ribera 2850, Concepción 4090541, Chile; gbcifuentess@gmail.com; 8 Faculty of Health and Social Sciences, Universidad de Las Américas, Santiago 8370040, Chile; alej.suazo@gmail.com; 9 GIAVAL Research Group, Department of Anatomy and Human Embryology, Faculty of Medicine, University of Valencia, 46001 Valencia, Spain; juan.sanchis@uv.es; 10 Faculty of Education, Universidad Autónoma de Chile, Santiago 8910060, Chile; hector.gutierrez@uautonoma.cl; 11 Departamento de Ciencias Química y Biológicas, Facultad de Ciencias de la Salud, Universidad Bernardo O’Higgins, Santiago 8370993, Chile

**Keywords:** vitamin D supplementation, vitamin D, diabetes mellitus type 1, diabetes type 2

## Abstract

**Background and Aims:** Numerous clinical and observational studies have examined the role of vitamin D in glycemic control and metabolic regulation among diabetic patients, but findings remain inconsistent. This meta-analysis aimed to assess the effects of vitamin D supplementation on glycosylated hemoglobin (HbA1c%), HOMA-IR, HOMA-β, LDL cholesterol, total cholesterol, triglycerides, fasting insulin, fasting plasma glucose, C-reactive protein, and the likelihood of reversion to normoglycemia in prediabetic individuals. **Methods:** A comprehensive search of multiple databases was performed using keywords including “diabetes mellitus,” “type 2 diabetes,” “vitamin D supplementation,” and “VD supplementation.” Twenty studies met the inclusion criteria. **Results:** Vitamin D supplementation was associated with significant improvements across several parameters, including HOMA-β (SMD = 0.71; 95% CI: 0.63–0.80; *p* < 0.00001), HDL cholesterol (SMD = 0.07; 95% CI: 0.05–0.09; *p* < 0.00001), and others (SMD = −0.40; 95% CI: −0.45 to −0.34; *p* < 0.00001). **Conclusions:** Vitamin D supplementation appears to provide beneficial effects on glycemic, lipid, and inflammatory markers in patients with diabetes and prediabetes. Specifically, supplementation significantly reduced HbA1c%, HOMA-IR, LDL cholesterol, total cholesterol, triglycerides, fasting insulin, fasting glucose, and C-reactive protein while increasing the rate of normoglycemia among prediabetic individuals. Further research is needed to strengthen the evidence base regarding vitamin D’s role in diabetes management.

## 1. Introduction

Vitamin D is an essential nutrient primarily synthesized through exposure to sunlight, as natural dietary sources are limited [1]. Significant food sources include fatty fish such as salmon, mackerel, and sardines, along with fortified dairy products and cereals [2]. In the body, vitamin D is converted to its active form, calcitriol, in the kidneys from its precursor 25-hydroxycholecalciferol, a process regulated by the parathyroid hormone (PTH). Calcitriol plays a vital role in calcium absorption and bone health while also exhibiting antioxidant, anti-inflammatory, and neuroprotective properties [3,4]. Optimal serum vitamin D levels are defined as greater than 30 ng/mL; concentrations between 21 and 29 ng/mL indicate insufficiency, and levels below 20 ng/mL signify deficiency [5]. The primary action of vitamin D occurs in the intestines, where it enhances calcium and phosphorus absorption by increasing calcium channel expression on the apical membrane of enterocytes, facilitating calcium transport into the bloodstream through ATP-dependent pumps. In bone tissue, vitamin D stimulates osteoblast differentiation and promotes the synthesis of osteocalcin and osteopontin, both essential for skeletal mineralization [6]. However, excessive intake can cause toxicity, leading to hypercalcemia and hyperphosphatemia, which may impair kidney function and damage soft tissues [7].

Diabetes mellitus (DM) is a metabolic disorder characterized by persistently elevated blood glucose levels. To understand this condition, it is essential to examine glucose metabolism and the role of insulin [7]. Glucose, the body’s primary energy source, is obtained through dietary intake and endogenous processes such as glycogen breakdown in the liver, stimulated by glucagon, and lipid mobilization from adipose tissue [8,9]. When blood glucose levels rise, pancreatic beta cells release insulin, which promotes glucose uptake into muscle and adipose tissues for immediate energy utilization or storage as glycogen and fat [9,10].

Diabetes occurs either due to insufficient insulin production or reduced cellular responsiveness to insulin, resulting in persistent hyperglycemia [11]. Type 1 diabetes (T1D) typically develops in children and adolescents and is caused by autoimmune destruction of pancreatic beta cells [12]. In contrast, type 2 diabetes (T2D) progresses gradually in adults and is often associated with insulin resistance, commonly linked to poor dietary habits and physical inactivity [13]. Uncontrolled diabetes can lead to symptoms such as blurred vision, fatigue, excessive thirst, frequent urination, increased appetite, and unexplained weight loss [13].

Vitamin D demonstrates potential therapeutic benefits, particularly in hereditary bone disorders, by enhancing calcitriol production, which promotes calcium and phosphate absorption [3]. It also works synergistically with calcium to lower the risk of osteoporosis and prevent bone mineral loss. The need for vitamin D increases with age, underscoring its role in maintaining overall health and metabolic balance. Evidence from some studies suggests that vitamin D supplementation may help lower cholesterol, reduce triglyceride synthesis, and improve high-density lipoprotein (HDL) levels. Additionally, it may inhibit renin activity, thereby decreasing cardiovascular risk by reducing activation of the renin–angiotensin–aldosterone system (RAAS), which plays a key role in the development of hypertension [14].

Vitamin D deficiency is defined as a serum concentration below 20 ng/mL, with levels under 12 ng/mL strongly associated with an elevated risk of osteometabolic disorders. Common contributing factors include inadequate sunlight exposure and poor dietary intake [14]. Clinical manifestations may include fatigue, muscle pain, and bone discomfort, as well as conditions such as rickets, osteomalacia, or osteoporosis, which result from impaired bone mineralization [2].

Due to its endocrine role and metabolic significance, fluctuations in vitamin D levels can substantially influence overall health outcomes [9,11,13]. Numerous studies have reported associations between vitamin D deficiency and both types of diabetes. Beyond its well-established role in skeletal health, vitamin D is essential for immune function and glucose metabolism, affecting both insulin secretion and sensitivity [14].

Vitamin D supports immune regulation by modulating inflammatory mediators and enhancing insulin receptor (INS-R) expression, thereby improving insulin sensitivity—a benefit particularly relevant in prediabetic individuals [5,7]. In type 1 diabetes (T1D), supplementation may offer beta-cell protection against autoimmune damage, potentially slowing the progressive decline in insulin secretion. Observational studies further suggest that insufficient vitamin D levels may increase the risk of developing T1D. Adequate vitamin D status also supports intestinal calcium absorption, influencing pancreatic beta-cell insulin release through calcium-dependent protein kinase C signaling pathways [15].

Given these findings, further investigation into the relationship between vitamin D and diabetes may provide valuable insights for developing targeted therapeutic strategies to manage this prevalent metabolic disorder.

## 2. Methods

### 2.1. Protocol and Registration

This systematic review and meta-analysis followed the Preferred Reporting Items for Systematic Reviews and Meta-Analyses (PRISMA) guidelines (see Supplementary Table S1) [16] and was registered in the International Prospective Register of Systematic Reviews (PROSPERO) under ID CRD420250655371.

### 2.2. Search

A comprehensive search was conducted across multiple electronic databases, including MEDLINE (via PubMed), EMBASE, Scopus, Cochrane Central Register of Controlled Trials, CINAHL, and Web of Science, covering all records up to February 2024. We included only randomized controlled clinical trials (RCTs) published in English or Spanish. The search terms included the following: “vitamin D supplementation” OR “Vitamin D” OR “Vitamin D supplement” OR Vitamin D supplements AND “diabetes” OR “impaired glucose tolerance” NOT “review” NOT “animals”. Two reviewers (RV & IC) independently screened titles and abstracts, retrieving full-text articles deemed relevant. Disagreements were resolved by a third reviewer (EB) (see Supplementary Table S2).

### 2.3. Study Selection

Eligible studies were RCTs involving participants with type 1 diabetes (T1DM) or type 2 diabetes (T2DM) who received varying doses of vitamin D supplementation. Studies evaluated biochemical, clinical, or inflammatory outcomes. Exclusions included letters, editorials, case reports, reviews, non-human studies, unrelated patient conditions, interventions unrelated to vitamin D, and trials without control groups (see Supplementary Table S3).

### 2.4. Risk of Bias Assessment

Two authors (AB & JJv-F) independently extracted data, including study characteristics (author, year, design), sample size, outcomes, dosage, administration methods, and geographic region. Risk of bias was assessed using the Cochrane Risk of Bias (RoB) tool [17], which evaluates seven domains: random sequence generation, allocation concealment, blinding, outcome assessment, incomplete data, selective reporting, and other biases. Disagreements were resolved through discussion with a third reviewer (JJV-F). Inter-reviewer agreement (kappa) was 0.91, indicating substantial agreement.

### 2.5. Data Synthesis and Statistical Analysis

Primary outcomes were extracted and analyzed using standardized units and scales. Key variables included the following:

- Anthropometric and biochemical markers: BMI (kg/m^2^), 25-hydroxy vitamin D [25(OH)D] (nmol/L), HbA1c (%), HDL and LDL cholesterol, creatinine (mg/dL), PTH (ng/dL), total cholesterol, triglycerides, fasting blood glucose, calcium and phosphorus (mmol/L), fasting insulin, and CRP (mg/L).
- Inflammatory markers: Interleukin-6 (IL-6) and Interleukin-1β (pg/mL).
- Blood pressure: systolic and diastolic (mmHg).
  Biomarkers were interpreted against clinical reference ranges:
- BMI: underweight (<18.5), normal (18.5–24.9), overweight (25–29.9), obese (>30).
- LDL: <130 mg/dL (optimal), >190 mg/dL (high risk).
- HDL: >40 mg/dL (males), >45 mg/dL (females).
- Total cholesterol: <200 mg/dL; triglycerides: <150 mg/dL.
- Waist circumference: ≥102 cm (males), ≥88 cm (females).
- Body fat: 8.1–15.9% (males), 15.1–20.9% (females).
- Normal fasting glucose: <100 mg/dL.

Effect sizes were calculated as mean differences (MD) and standardized mean differences (SMD) using Cohen’s d: trivial (<0.2), small (0.2–0.5), medium (0.6–0.8), large (>0.8). Pooled effect sizes with 95% confidence intervals (CI) were computed using the Mantel-Haenszel fixed-effect model, depending on heterogeneity. Heterogeneity (*I*^2^) was classified as follows: 0–40% (not important), 30–60% (moderate), 50–90% (substantial), and 75–100% (considerable). Forest plots were visually inspected for overlapping CIs. All analyses were performed using Review Manager (RevMan) version 5.4.

### 2.6. Quality of Evidence

The certainty of evidence for each outcome was assessed using the GRADE system, which categorizes evidence as being of high, moderate, low, or very low quality [18]. Data from RevMan were imported into GRADEpro to generate the “Summary of Findings” table (see Supplementary Table S3).

## 3. Results

### 3.1. Study Selection

**Figure 1:**
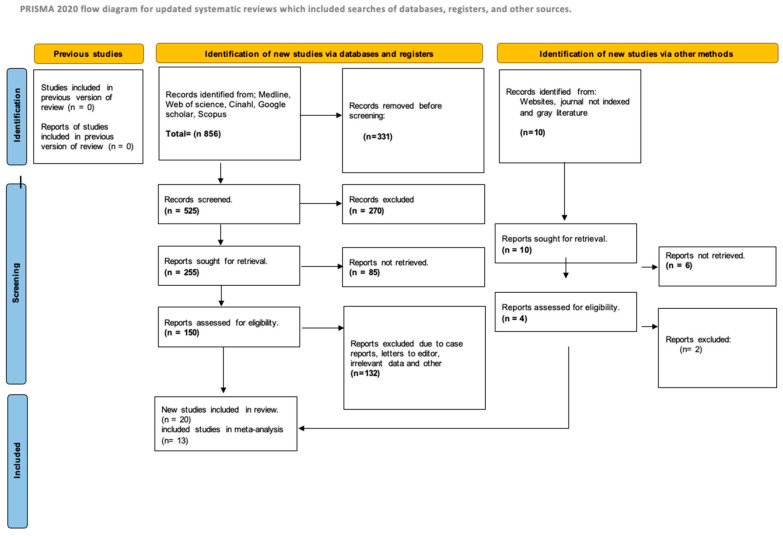
Diagram of the search workflow.

The database search yielded 478 articles, of which 147 full-text studies were reviewed for eligibility. The selection process is illustrated in Figure 1 (PRISMA flowchart). No additional studies were identified from clinical trial registries. Ultimately, 20 RCTs met inclusion criteria for the systematic review [10,11,12,13,14,15,16,17,18,19,20,21,22,23,24,25,26,27,28,29,30,31,32,33,34,35,36,37,38]. Of these, nine meta-analyses addressing the effects of vitamin D on various outcomes incorporated 13 studies into the quantitative synthesis [18,21,24,29,35,39,40,41,42,43,44,45,46]. The reasons for study exclusion are detailed in Supplementary Table S2.

### 3.2. Study Characteristics

A summary of the included studies is presented in Table 1. The eligible studies assessed the effects of vitamin D administered in various forms, including drops, capsules, tablets, standalone supplements, and multivitamins. These studies, published between 2013 and 2024, were conducted in multiple countries, including the USA, Korea, Norway, Canada, India, Iran, Denmark, Lebanon, China, UAE, Russia, Japan, Serbia, and the UK. Several studies employed multicenter designs, thereby expanding the geographical diversity of the research.

The combined sample across all studies included 14,831 participants, of whom 8196 were assigned to the vitamin D supplementation group and 6635 to comparison groups receiving alternative interventions. The mean age of participants across both groups was 50.89 years, with an average follow-up period of 17 months.

For the meta-analysis, 13 of the 20 studies [18,21,24,29,35,39,40,41,42,43,44,45,46] were included based on comparable follow-up durations, resulting in a pooled sample of 3893 participants with a mean age of 47.9 years.

**Table 1:** Characteristics of the included studies.

Author	Country		Population	Intervention		Outcomes	Follow-Up	Results
		Sample Size (*n*)	Patients Mean Age (SD)	Type of Intervention	Characteristics and Doses			
Cojic et al., 2021 [20]	Serbia	CG: 65 EG:49	≥30	CG: Metformin EG: Metformin + Vitamin D3	CG: 65 patients with T2DM were given metformin alone as a control treatment. EG: 49 patients with T2DM were given metformin and vitamin D3 as treatment. In total, 33 patients were given 50,000 IU/day (14 drops) of vitamin D3 for the first 3 months and then 14,000 IU/day (4 drops) for another 3 months. The other 16 patients were given 14,000 IU/day (4 drops) for the next 6 months. All patients were given their corresponding metformin	Vitamin D (nmol/L) HbA1c (%) IF (mU/L) BMI (kg/m^2^) WC (cm) HOMA-IR FBG (mmol/L) SBP (mmHg) DBP (mmHg) MDA (TBARS) (mM/L) AOPP (μM chloramine T equivalents) CRP (mg/L) TC (mmol/L) TG (mmol/L) HDL (mmol/L) LDL (mmol/L) Castelli I TG/TBARS AC Ca++	6 months	6 months Vitamin D (nmol/L) *p* = <0.001 HbA1c (%) *p* = 0.045 IF (mU/L) *p* = 0.245 BMI (kg/m^2^) *p* = 0.288 WC (cm) *p* = 0.086 HOMA-IR *p* = 0.203 FBG (mmol/L) *p* = 0.116 SBP (mmHg) *p* = 0.373 DBP (mmHg) *p* = 0.299 MDA (TBARS) (mM/L) *p* = 0.215 AOPP (μM chloramine T equivalents) *p* = 0.776 CRP (mg/L) *p* = 0.385 TC (mmol/L) *p* = 0.756 TG (mmol/L) *p* = 0.156 HDL (mmol/L) *p* = 0.270 LDL (mmol/L) *p* = 0.209 Castelli I *p* = 0.409 TG/TBARS *p* = 0.288 AC *p* = 0.333 Ca++ *p* = 0.874
Boer et al., 2019 [21]	USA	CG: 320 EG1: 370 EG2: 333 EG3: 289	67.6	CG: 2 Placebos EG1: Omega 3 fatty acids + Vitamin D3 EG2: Vitamin D3 + Placebo EG3: Omega 3 fatty acids + Placebo	CG: 320 patients with type 2 diabetes were given two inert placebos. EG1: 370 patients with type 2 diabetes were administered vitamin D3 (cholecalciferol, 2000 IU) together with omega-3 fatty acids (fish oil, 1 g capsules containing 465 mg eicosapentaenoic acid [EPA] plus 375 mg docosahexaenoic acid [DHA]). EG2: 333 patients with type 2 diabetes were administered Vitamin D3 (cholecalciferol, 2000 IU) together with an inert placebo. EG3: 289 patients with type 2 diabetes were administered omega-3 fatty acids (fish oil, 1 g capsules containing 465 mg eicosapentaenoic acid [EPA] plus 375 mg docosahexaenoic acid [DHA]) together with inert placebo	Vitamin D Omega-3 fatty acids	2 years and 9 months	Vitamin D *p* = 0.25 Omega-3 Fatty acids *p* = 0.27
Byrn et al., 2022 [32]	USA	CG: 15 EG:15	CG: 55.62 EG: 55.80	CG: Vitamin D3 supplement in a ten times lower dose EG: High-dose Vitamin D3 supplement	CG: received a low-dose Vitamin D3 (Cholecalciferol) therapy of 5000 IU for 12 weeks EG: they received a therapy of a weekly supplement of vitamin D3 (Cholecalciferol) in high doses of 50,000 IU for 12 weeks	Symbol-Digit Modality Z-score Symbol-Digit Modality Z-score Verbal fluency Z-score HVLT total recall T-score HVLT delayed recall T-score HVLT retention T-score Stroop word reading Z-score Stroop color naming Z-score Stroop Interference Z-score Trail Making Test Part A Z-score	3 months	Symbol-Digit Modality Z-score *p* value not reported Symbol-Digit Modality Z-score *p* value not reported Symbol-Digit Modality Z-score *p* value not reported Verbal fluency Z-score *p* value not reported HVLT total recall T-score *p* value not reported HVLT delayed recall T-score *p* value not reported HVLT retention T-score *p* value not reported Stroop word reading Z-score *p* value not reported Stroop color naming Z-score *p* value not reported Stroop Interference Z-score *p* value not reported Trail Making Test Part A Z-score *p* value not reported
Huang et al., 2021 [17]	China	CG:70 EG:80	CG: 31.3 ± 4.7 EG:31.5 ± 4.1	CG: placebo EG: intake of Vitamin D and omega-3 fatty acids.	CG: placebo EG: the test group took 40,000 IU of vitamin D and 8000 mg of omega-3 fatty acids twice daily	FBG (mmol/L) Fasting insulin (ulU/mL) HOMA-IR HOMA-β TGs (mmol/L) Total cholesterol (mmol/L) LDL (mmol/L) HDL (mmol/L) VLDL (mmol/L)	6 weeks	FBG (mmol/L) *p* ≤ 0.001 Fasting insulin (ulU/mL) *p* ≤ 0.001 HOMA-IR *p* ≤ 0.001 HOMA-β *p* ≤ 0.001 TGs (mmol/L) *p* ≤ 0.001 Total cholesterol (mmol/L) *p* ≤ 0.001 LDL (mmol/L) *p* ≤ 0.001 HDL (mmol/L) *p* = 0.89 VLDL (mmol/L) *p* = 0.008
Chou et al., 2021 [18]	USA	CG: 383 EG: 388	CG: 63.9 EC: 63.7	CG: Placebo EG: Vitamin D3 supplement and/or Omega-3 fatty acid d	CG: placebo EG: vitamin D3 supplement at a dose of 2000 IU per day and/or Omega-3 fatty acid 1 g per day	Weight, mean (SD), kg BMI, mean (SD), kg/m^2^ Waist circumference, mean (SD), cm % Body fat, mean (SD) IMF, mean (SD), kg/m^2^ Fat to lean mass ratio, mean (SD) VAT area, mean (SD), cm^2^ Truncal fat mass, mean (SD), kg Truncal-to-limb fat ratio, mean (SD) LMI, mean (SD), kg/m^2^ ALM, mean (SD), kg ALM/BMI, mean (SD)	2 years	Weight, mean (SD), kg *p* = 0.76 BMI, mean (SD), kg/m^2^ *p* = 0.83 Waist circumference, mean (SD), cm *p* = 0.43 % Body fat, mean (SD) *p* = 0.93 IMF, mean (SD), kg/m^2^ *p* = 0.92 Fat to lean mass ratio, mean (SD) *p* = 0.83 VAT area, mean (SD), cm^2^ *p* = 0.58 Truncal fat mass, mean (SD), kg *p* = 0.69 Truncal-to-limb fat ratio, mean (SD) *p* = 0.29 LMI, mean (SD), kg/m^2^ *p* = 0.19 ALM, mean (SD), kg *p* = 0.89 ALM/BMI, mean (SD) *p* = 0.43
Angellotti et al., 2019 [23]	USA	CG:61 EG:66	average of 60 years	CG: Placebo EG: pill with 4000 IU of Vitamin D3 (cholecalciferol).	CG: they were given 1 placebo pill daily. EG: they were given a daily pill with 4000 IU of vitamin D3 (cholecalciferol)	Total cholesterol, mg/dL HDL, mg/dL TG, mg/dL TG/HDL ratio LDL, mg/dL C-reactive protein, mg/L CVD risk	48 weeks	Total Cholesterol, mg/dL *p* = 0.666 HDL, mg/dL *p* = 0.323 TG, mg/dL *p* = 0.032 TG/HDL ratio *p* = 0.056 LDL, mg/d *p* = 0.99 C-reactive protein, mg/L *p* = 0.774 CVD risk *p* = 0.297
Gnudi et al., 2023 [24]	UK	CG: 30 EG:25	>40 years	CG: placebo EG: Calcitriol	CG: they were given placebo EG: they were given 0.5 mcg of Calcitriol per day	LVMI (g/m^2^) Interstitial myocardial fibrosis (% CVD) Left ventricular end systolic volume (mL/m^2^) Left ventricular end diastolic volume (mL/m^2^) Left ventricular ejection fraction (%)	9 years	LVMI (g/m^2^) *p* = 0.24 Interstitial myocardial fibrosis (% CVD) *p* = 0.09 Left ventricular end systolic volume (mL/m^2^) *p* = 0.42 Left ventricular end diastolic volume (mL/m^2^) *p* = 0.28 Left ventricular ejection fraction (%) *p* = 0.84
Huang et al., 2013 [26]	China	2708	48.5 ± 12.6 years	Q1:677 Q2:677 Q3:677 Q4:677	The patients in the study were separated into quartiles based on lipoprotein lipase levels, obtaining Q1 (<532.8), Q2 (532.9−653.2), Q3 (653.3–778.6), Q4 (>778.6)	25(OH)D, ng/mL GFR, mmol/L PG, mmol/L HbAlc, % Insulin, mU/L HOMA-IR TC, mmol/L TG, mmol/L HDL-C, mmol/L LDL-C, mmol/L apoA, mmol/L apoB, mmol/L FFAs, μmol/L	One shot	25(OH)D, ng/mL *p* < 0.001 GFR, mmol/L *p* = 0.021 PG, mmol/L *p* = 0.008 HbAlc, % *p* < 0.001 Insulin, mU/L *p* = 0.149 HOMA-IR *p* < 0.001 TC, mmol/L *p* = 0.201 TG, mmol/L *p* < 0.001 HDL-C, mmol/L *p* = 0.032 LDL-C, mmol/L *p* = 0.068 apoA, mmol/L *p* < 0.001 apoB, mmol/L *p* = 0.470 FFAs, μmol/L *p* < 0.001
Yin et al., 2024 [28]	China	CG: 771 EG: 766	28.5 years average	CG: 400 IU/d vitamin D3 EG: 1600 IU/d vitamin D3	CG: serum 25(OH)D concentration <75 nmol/L at a dose of 400 IU/day EG: serum 25(OH)D concentration <75 nmol/L at a dose of 1600 IU/day	HDLC, mmol/L LDLC, mmol/L TC, mmol/L TG, mmol/L CRP, mg/L TNF-a, pg/mL Il-6, pg/mL IL-1β, pg/mL E-Selectin, ng/mL ICAM, ng/mL SBP, mmHg DBP, mmHg TyG	2 months	HDLC, mmol/L *p* = 0.01 LDLC, mmol/L *p* = 0.69 TC, mmol/L *p* = 0.20 TG, mmol/L *p* = 0.35 CRP, mg/L *p* = 0.11 TNF-a, pg/mL *p* = 0.09 Il-6, pg/mL *p* = 0.26 IL-1 β, pg/mL *p* = 0.45 E-Selectin, ng/mL *p* = 0.28 ICAM, ng/mL *p* = 0.17 SBP, mmHg *p* = 0.25 DBP, mmHg *p* = 0.32 TyG *p* = 0.61
Rasouli et al., 2022 [29]	USA	CG: 886 EG: 888	60.5 ± 9.8 years	CG: placebo equivalent EG: 4000 IU of vitamin D3 (cholecalciferol)	CG: participants were asked to refrain from using specific diabetes or weight-loss medications during the study and to limit off-study vitamin D use to 1000 IU per day from all supplements, including multivitamins. Follow-up visits were carried out at months 12 and 24 EG: Participants were asked to refrain from using specific diabetes or weight-loss medications during the study and to limit off-study vitamin D use to 1000 IU per day from all supplements, including multivitamins. Follow-up visits were carried out at months 12 and 24	CPI IGI DI	At the beginning, at month 12 and at month 24	BASELINE 0.64 MONTH 12 0.61 MONTH 24 0.995
Riek et al., 2018 [30]	USA	CG: 15 EG: 11	CG: 57.6 ± 1.9 EG: 57.4 ± 1.8	CG: placebo GA: 25(OH)D < 25 ng/mL randomly assigned to vitamin D3 4000 IU daily	Subjects were randomly assigned 1:1 to one of two groups: vitamin D3 4000 international units (IU) or matching placebo daily (supplied by Tishcon Corp.) for 4 months, with treatment allocation blinded to both investigators and participants. Both groups received calcium carbonate 500 mg twice daily. For safety reasons, patients They were examined at 2 weeks, 1 month, 2 months, 3 months and 4 months for evaluation of blood levels	BMI (kg/m^2^) Total cholesterol LDL HDL Triglycerides Hemoglobin A1c	They were observed at 2 weeks, 1 month, 2 months, 3 months and 4 months.	BMI (kg/m^2^) = 0.06 Total cholesterol = 0.61 LDL = 0.70 HDL = 0.79 Triglycerides = 0.36 Hemoglobin A1c = 0.81
Pittas et al., 2019 [31]	USA	CG: 1212 placebo EG: 1211 vitamin D group		EG: 4000 IU of vitamin D3 CG a corresponding placebo.	Participants were asked to refrain from using specific diabetes or weight-loss medications during the trial and to limit their off-trial vitamin D use to 1000 IU per day from all supplements, including multivitamins. Participants were asked to limit calcium supplements to 600 mg per day. Participants were followed up for 4 years	Fasting plasma glucose 2 h post-load plasma glucose Glycated hemoglobin Serum 25-hydroxyvitamin D	Month 3, month 6 and twice a year thereafter until year 4	Does not report
Penckofer et al., 2022 [32]	USA	CG: EG:	50.58 (11.13)	EG: 50,000 IU of calciferol weekly EG: 5000 IU of calciferol weekly	A total of 119 women (57 at the lowest dose and 62 at the highest dose) received weekly oral vitamin D3 supplements (50,000 IU) or an active comparator (5000 IU) for 6 months. Vitamin D, 25-hydroxyvitamin D [25(OH)D] levels, and depression were measured at baseline, 3, and 6 months.	Vitamin D laboratory values (ng/mL) Serum 25(OH)D Creatinine Mean systolic blood pressure Mean diastolic blood pressure Average body mass index Average HbA1c Average fasting blood glucose	Follow-up at 3 and 6 months. Women were contacted by telephone (2 months and 4 and 5 months) to assess depressive symptoms and adverse events	Laboratory values of serum vitamin D (ng/mL) 25 (OH) D: *p* = 0.06 Creatinine Mean systolic blood pressure Mean diastolic blood pressure Average body mass index Average HbA1c Average fasting blood glucose
Kawahara et al., 2022 [33]	JAPAN	CG: 626 EG:630	61.3 years.	EG: 75 μg of eldecalcitol CG: placebo equivalent.	Participants were randomly assigned to take a single hard gel tablet, once a day, containing 75 μg of eldecalcitol or a matching placebo, which has the same appearance	Glycated hemoglobin fasting plasma glucose concentration Plasma glucose concentration two hours after loading Random plasma glucose concentration	Three-month intervals, and the follow-up period concluded after three years	Primary outcome (*p* = 0.39) Secondary outcomes *p* = 0.020
Karonova et al., 2020 [34]	RUSSIA	GC: 34 EG:33	Average age 56 (49; 61)	GC: 5000 IU once a week EG: 40,000 IU	Patients were randomly assigned by odd/even method into two cholecalciferol treatment groups: Group I (*n* = 34) 5000 IU once weekly and Group II (*n* = 33) 40,000 IU once weekly, orally for 24 weeks	25(OH)D—25-hydroxyvitamin D HbA1c—glycated hemoglobin; PTH—parathyroid hormone TC—total cholesterol TNFα—tumor necrosis factor α CRP—C-reactive protein IL-1β—interleukin 1β IL-6—interleukin-6 IL-10—interleukin-10	24 weeks	HbA1c (*p* = 0.031) IL-6 (*p* = 0.017) IL-10 (*p* = 0.030)
Johny et al., 2022 [35]	INDIA	CG:placeBO 29 EG:Vitamin D3 30	CG: 55.06 ± 9.57 EG: 53.6 ± 9.6	EG: 60,000 IU of cholecalciferol/week, control dose 60,000 IU/month CG: Placebo equivalent (powdered starch compound)	The vitamin D3 group received 60,000 IU of cholecalciferol/week for the initial 3 months as a control dose, followed by 60,000 IU/month for 3 months as a maintenance dose. The placebo group received a matching placebo (consisting of powdered starch) similar to vitamin D	HbA1c Total 25-OH vitamin D (ng/mL) Duration of type 2 diabetes (years) Total cholesterol (mg/dL) Triglycerides (mg/dL) HDL (mg/dL) LDL (mg/dL) Uric acid (mg/dL) Creatinine (mg/dL)	6 months	*p* < 0.05
Chao Gu et al., 2022 [36]	China	CG:92 EG:86	Does not report	CG: received regular treatment (type 2 diabetes group) EG: They received an additional 400 IU of vitamin D per day	Patients with type 2 diabetes were randomly assigned to receive an additional vitamin D supplement (*n* = 86) or not (*n* = 92) in addition to standard drug treatments	Vitamin D GSH Metabolic enzyme GSH GCLC GR Inflammatory factor MCP-1 IL-8	90 days	*p* < 0.05
Sadiya et al., 2015 [37]	United Arab Emirates	CG: 42 EG: 45	CG: 48 ± 8 EG: 49 ± 8	CG: They received starch capsules EG: 3000 IU/day of oral vitamin D	It was divided into two phases of 3 months each. In phase 1, group D (*n* = 45) received unlabeled oral vitamin D at 6000 IU/day, while group P (*n* = 42) received placebo capsules. In phase 2, group D (*n* = 45) received 3000 IU/day of oral vitamin D and group P (*n* = 42) continued on matching placebo capsules. Participants were advised to maintain their usual medical care and diets and to avoid taking calcium or vitamin D supplements on their own during the study period	yeah	6 months	Does not report
Peivasteh Safarpour et al., 2020 [38]	Iran	CG: 43 EG: 42	CG: 50.05 ± (10.7) EG: 50.36 ± (10.2)	CG: or took similar pearls containing oral paraffin without VD 50,000 IU/week EG: took 8 pearls of VD 50,000 IU/week	At baseline and endpoint, demographic, anthropometric, and dietary intake characteristics were determined using two 24 h food recalls (one weekend and one weekday). Physical activity was measured using a short version of the International Physical Activity Questionnaire (IPAQ). Sun exposure status was assessed using a valid questionnaire. Blood was drawn from the brachial vein at baseline and endpoint for measurement of serum factors	Vitamin D (ng/mL) HbA1c (%) SIRT1 (ng/mL) Irisin (ng/mL) HOMA-IR (N) QUICKI (N)	8 weeks	GL: Vitamin D (ng/mL) < 0.001 HbA1c (%) 0.657 SIRT1 (ng/mL) < 0.001 Irisin (ng/mL) < 0.001 HOMA-IR (N) 0.003 FAST (N) 0.003 GA: Vitamin D (ng/mL) < 0.001 HbA1c (%) < 0.001 SIRT1 (ng/mL) < 0.001 Irisin (ng/mL) < 0.001 HOMA-IR (N) 0.006 FAST (N) 0.005
Xiaomi Sun et al., 2023 [39]	China	CG: 15 EG: 46	50.1 years ± 7.3 years	They received an exercise program and a daily dose of 1000 IU of vitamin D. They only performed the exercise program without vitamin D supplementation. They received a daily dose of 10,000 IU of vitamin D3. They received a placebo without exercise or vitamin D.	This was a randomized controlled trial (RCT) with a 12-week intervention, followed by an additional 12-week follow-up period. In the 12-week intervention, participants in the intervention groups (EX + VD and VD) received vitamin D supplementation and/or exercise, while the control group received a placebo. After the intervention, follow-up was conducted to assess the persistence of the intervention’s effects on participants. This is important to understand whether the observed benefits are maintained over the long term	table page 8	12 weeks	Does not report
Shih et al., 2014 [47]	USA	CG:12 EG:13	<18 years old	EG: Vitamin D3 for 6 months CG: no treatment for 6 months and then vitamin D3 treatment for another 6 months	EG: therapy with 20,000 IU of Vitamin D3 per week for 6 months followed by 6 months of observation CG: 6 months of observation without clinical intervention and then 6 months of treatment with 20,000 IU per week	BMI (kg/m) + SD Normal weight (<85th %ile) Overweight/obese (>85%) Systolic BP (mmHg) + SD Diastolic BP (mmHg) + SD Type 1 diabetes duration (yr) + SD Type of insulin − number (%) Lispro insulin Aspart insulin HbA1c (%) + SD HbA1c (mmol/mol) Total daily insulin dose + SD (units/kg/day 25-OH vitamin D (ng/mL) + SD CRP (mg/dL) IL-6 (pg/mL) TNF-a (pg/mL)	6 months	BMI (kg/m) + SD *p* = 0.037 Normal weight (<85th %ile) *p* = 0.09 Overweight/obese (>85%) Systolic BP (mmHg) + SD *p* = 0.66 Diastolic BP (mmHg) + SD *p* = 0.23 Type 1 diabetes duration (yr) + SD *p* = 0.34 Type of insulin − number (%) *p* = 0.30 Lispro insulin Aspart insulin HbA1c (%) + SD *p* = 0.004 HbA1c (mmol/mol) Total daily insulin dose + SD (units/kg/day) *p* = 0.76 25-OH vitamin D (ng/mL) + SD *p* = 0.0001 CRP (mg/dL) *p* = 0.85 IL-6 (pg/mL) *p* = 0.026 TNF-a (pg/mL) *p* = 0.16
Corbin et al., 2023 [48]	USA	CG:1212 EG:1211	does not provide information	CG: Placebo EG: soft gel tablet once a day containing 4000 IU of vitamin D3	CG: 1 placebo pill per day EG: soft gel tablet once a day containing 4000 IU of vitamin D3	25-hydroxyvitamin D Level (ng/mL) NAFLD-Liver Fat Score Fibrosis-4 Score AST to Platelet Ratio Index	2.5 years	25-hydroxyvitamin D Level (ng/mL) *p* = 0.005 NAFLD-Liver Fat Score *p* ≤ 0.001 Fibrosis-4 Score *p* = 0.310 AST to Platelet Ratio Index *p* = 0.286
El Hajj et al., 2020 [49]	Lebanon	CG:43 EG:45	66.3 (±4.4) years	CG: Placebo Pill EG: Vitamin D supplement	CG: placebo pill three times a week for 6 months EG: received 30,000 IU of cholecalciferol per week (three doses of 10,000 IU per week) for a period of 6 months	25(OH)D (ng/mL) BMI (kg/m^2^) Waist circumference (cm) Body fat (%) Systolic BP (mmHg) Diastolic BP (mmHg) TG (mg/dL) TC (mg/dL) HDL-C (mg/dL) LDL-C (mg/dL) FBG (mg/dL) HbA1c (%) HOMA-IR PTH (ng/L)	6 months	25(OH)D (ng/mL) *p* < 0.0001 BMI (kg/m^2^) *p* < 0.0001 Waist circumference (cm) *p* = 0.0001 Body fat (%) *p* = 0.05 Systolic BP (mmHg) *p* = 0.34 Diastolic BP (mmHg) *p* = 0.21 TG (mg/dL) *p* = 0.02 TC (mg/dL) *p* = 0.38 HDL-c (mg/dL) *p* = 0.022 LDL-c (mg/dL) *p* = 0.18 FBG (mg/dL) *p* = 0.84 HbA1c (%) *p* = 0.31 HOMA-IR *p* = 0.26 PTH (ng/L) *p* < 0.0001
Gulseth et al., 2017 [40]	Norway	CG: 25 EG: 28	average age 55.7 ± 9.5 years	CG: Placebo EG: Vitamin D Supplement	CG: they received a single dose of oral placebo EG: they received a single dose of 400,000 IU oral vitamin D3 (received an additional 200,000 IU of vitamin D3 if serum 25(OH)D is <100 nmol/L after 4 weeks)	HbA1c (%) Fasting insulin (mmol/L) Fasting C-peptide (pmol/L) AIRg 0–8 DC-peptide max Glucose infusion rate (mmol/kg FFM/min) Total Rd (mmol/kg FFM/min) Basal EGP (mmol/kg FFM/min) Clamp EGP (mmol/kg FFM/min) Basal glucose oxidation (mmol/kg FFM/min) Basal nonoxidative glucose consumption (mmol/kg FFM/min) Basal fat oxidation (mg/kg FFM/min) Clamp glucose oxidation (mmol/kg FFM/min) Clamp nonoxidative glucose consumption (mmol/kg FFM/min) Clamp fat oxidation (mg/kg FFM/min) Resting energy expenditure (kcal/day) Clamp energy expenditure (kcal/day)	6 months	HbA1c (%) *p* = 0.98 Fasting insulin (mmol/L) *p* = 0.38 Fasting C-peptide (pmol/L) *p* = 0.73 AIRg 0–8 *p* = 0.10 DC-peptide max *p* = 0.04 Glucose infusion rate (mmol/kg FFM/min) *p* = 0.68 Total Rd (mmol/kg FFM/min) *p* = 0.52 Basal EGP (mmol/kg FFM/min) *p* = 0.37 Clamp EGP (mmol/kg FFM/min) *p* = 0.17 Basal glucose oxidation (mmol/kg FFM/min) *p* = 0.88 Basal nonoxidative glucose consumption (mmol/kg FFM/min) *p* = 0.66 Basal fat oxidation (mg/kg FFM/min) *p* = 0.61 Clamp glucose oxidation (mmol/kg FFM/min) *p* = 0.47 Clamp nonoxidative glucose consumption (mmol/kg FFM/min) *p* = 0.87 Clamp fat oxidation (mg/kg FFM/min) *p* = 0.20 Resting energy expenditure (kcal/day) *p* = 0.31 Clamp energy expenditure (kcal/day) *p* = 0.82
Joergensen et al., 2014 [41]	Denmark	CG:23 EG:22	average age 57 years	CG: Placebo capsule followed by paricalcitol capsule EG: Paricalcitol capsule followed by placebo capsule	CG: they received a daily placebo capsule for an initial 12 weeks, followed by a daily paricalcitol capsule for the subsequent 12 weeks EG: they received one capsule of paricalcitol daily for 12 weeks followed by one capsule of placebo daily for the next 12 weeks	Plasma *N*-terminal proBNP, pmol/L Plasma MR-proANP, pmol/L Plasma copeptin, pmol/L Urinary albumin excretion rate mg/24 h Estimated GFR mL/min/1.73 m^2^ GFR mL/min/1.73 m^2^	24 weeks	Plasma *N*-terminal proBNP, pmol/L *p* = 0.39 Plasma MR-proANP, pmol/L *p* = 0.57 Plasma copeptin, pmol/L *p* = 0.14 Urinary albumin excretion rate mg/24 h *p* = 0.03 Estimated GFR mL/min/1.73 m^2^ *p* = 0.012 GFR ml/min/1.73 m^2^ *p* = 0.2
Limonte et al., 2021 [42]	USA	CG: 320 EG1: 333 EG2: 289 EG3: 370	67.6 years average	CG: Vitamin D3 placebo and *n*-3 fatty acid placebo EG1: Vitamin D3 and *n*-3 fatty acid placebo EG2: *n*-3 fatty acid and vitamin D3 placebo EG3: Vitamin D3 and *n*-3 fatty acid	CG: daily administration of placebos compatible with Vitamin D3 and placebo compatible with *n*-3 fatty acid EG1: daily administration of Vitamin D3 (2000 IU) and daily placebo compatible with *n*-3 fatty acid EG2: daily administration of *n*-3 fatty acid (1 g) and daily placebo compatible with Vitamin D3 EG3: daily administration of Vitamin D3 (2000 IU) and daily administration of *n*-3 fatty acid (1 g)	IL-6 (pg/mL) hsCRP (mg/L) NT-proBNP (ng/L)	5 years	IL-6 (pg/mL) *p* = 0.38 hsCRP (mg/L) *p* = 0.33 NT-proBNP (ng/L) *p* = 0.0034
Tabesh et al., 2014 [50]	Iran	CG: 30 EG1: 29 EG2: 29 EG3: 30	>30 years	CG: Vitamin D placebo and Calcium placebo EG1: Vitamin D supplement and Calcium Placebo EG2: Calcium supplement and Vitamin D placebo EG3: Vitamin D and Calcium supplements	CG: received separate placebos of calcium and vitamin D3 EG1: received 50,000 U of vitamin D3 supplements weekly along with daily Calcium Placebo EG2: received a 1000 mg calcium carbonate supplement daily along with a weekly vitamin D3 placebo EG3: received 50,000 U of vitamin D3 supplements weekly along with 1000 mg of calcium carbonate supplements daily	FPG (mmol/L) HbA1c (%) Insulin (pmol/L) HOMA-IR QUICKI HOMA-β TG (mmol/L) HDL-C (mmol/L) LDL-C (mmol/L) TC (mmol/L) TC/HDL-C Non-HDL-C (mmol/L)	8 weeks	FPG (mmol/L) *p* = 0.12 HbA1c (%) *p* = 0.61 Insulin (pmol/L) *p* = 0.16 HOMA-IR *p* = 0.54 QUICKI *p* = 0.02 HOMA-β *p* = 0.04 TG (mmol/L) *p* = 0.77 HDL-C (mmol/L) *p* = 0.11 LDL-C (mmol/L) *p* = 0.21 TC (mmol/L) *p* = 0.001 TC/HDL-C *p* < 0.001 Non-HDL-C (mmol/L) *p* < 0.001
Upreti et al., 2018 [43]	India	CG:30 EG:30	CG: 49.9 ± 6.9 EG:48.3 ± 9.8	CG: Placebo EG: Vitamin D supplementation	CG: oral placebo (microcrystalline cellulose), both drugs were administered in identical containers. EG: they received oral vitamin D (calcirol 60,000 IU each week for the first six weeks and then once every 4 weeks weeks until the end of the study)	Vitamin D (ng/mL) FPG (mg/dL) PPPG (mg/dL) HbA1c (%) Systolic BP (mmHg) Diastolic BP (mmHg) Total cholesterol (mg/dL) Triglycerides (mg/dL) HDL cholesterol (mg/dL) LDL cholesterol (mg/dL)	6 months	Vitamin D (ng/mL) *p* = <0.001 FPG (mg/dL) *p* = <0.001 PPPG (mg/dL) *p* = <0.001 HbA1c (%) *p* = 0.006 Systolic BP (mmHg) *p* = 0.002 Diastolic BP (mmHg) *p* = 0.03 Total cholesterol (mg/dL) *p* ≤ 0.001 Triglycerides (mg/dL) *p* = 0.39 HDL cholesterol (mg/dL) *p* = 0.17 LDL cholesterol (mg/dL) *p* = 0.05
Mager et al., 2016 [44]	Canada	Group 1: 60 Group 2: 60	From 18 to 80 years old.	Group 1: Daily Vitamin D3 Group 2: Monthly vitamin D3	Group 1: daily dose of 2000 IU/D of vitamin D3 Group 2: monthly dose of 40,000 IU of vitamin D3	Hemoglobin Alc, % Blood Glucose, mmol/L Creatinine, u mol/L Urea, mmol/L ACR, mg/mmol Albumin g/L Calcium, mmol/L Phosphorus, mmol/L Magnesium, mmol/L PTH, pmolL ALP4, U/L FGF-234 pg/mL	6 months	Hemoglobin Alc, % *p* = 0.69 Blood Glucose, mmol/L *p* = 0.10 Creatinine, u mol/L *p* = 0.91 Urea, mmol/L *p* = 0.83 ACR, mg/mmol *p* = 0.20 Albumin g/L *p* = 0.69 Calcium, mmol/L *p* = 0.94 Phosphorus, mmol/L *p* = 0.92 Magnesium, mmol/L *p* = 0.93 PTH, pmolL *p* = 0.97 ALP4, U/L *p* = 0.77 FGF-234 pg/mL *p* = 0.36
Ryu et al., 2014 [45]	Korea	CG:30 EG:32	30 to 69 years old.	CG: placebo EG: Vitamin D	CG: placebo containing 100 mg of elemental calcium twice daily EG: 1000 IU of cholecalciferol (inactive vitamin D3, Dalim BioTech, Hwaseong, Korea) combined with 100 mg of elemental calcium (vitamin D group) twice a day	HbA1c, % Fasting glucose, mg/dL HOMA-IR 25(0H)D, ng/mL Total cholesterol, mg/dL HDL-C, mg/dL Triglyceride, mg/dL LDL-C, mg/dL Calcium, mg/dL Phosphorus, mg/dL PTH, pg/mL hsCRP, mg/dL pSBP, mmHg pDBP, mmHg CSBP, mmHg AIx,% baPWV, cm/s? OA, h	24 weeks	HbA1c, % *p* = 0.280 Fasting glucose, mg/dL *p* = 0.919 HOMA-IR *p* = 0.981 25(0H)D, ng/mL *p* = <0.001 Total cholesterol, mg/dL *p* = 0.248 HDL-C, mg/dL *p* = 0.998 Triglyceride, mg/dL *p* = 0.682 LDL-C, mg/dL *p* = 0.092 Calcium, mg/dL *p* = 0.768 Phosphorus, mg/dL *p* = 0.323 PTH, pg/mL *p* = 0.133 hsCRP, mg/dL *p* = 0.261 pSBP, mmHg *p* = 0.948 pDBP, mmHg *p* = 0.970 CSBP, mmHg *p* = 0.815 AIx,% *p* = 0.399 baPWV, cm/s *p* = 0.348 OA, h *p* = 0.990

CPI, C-peptide index; DI, disposition index using insulin-based indices; IGI, insulinogenic index; BMI: body mass index; CG: control group; EG: experimental group; EWL: excess weight loss; TWL: total weight loss; T2DM: type 2 diabetes mellitus; HbA1c, glycated hemoglobin; FI, fasting insulin; BMI, body mass index; toilet, waist circumference; HOMA-IR, Homeostatic Model Assessment of Insulin Resistance; FBG, fasting blood glucose; SBP, systolic blood pressure; DBP, diastolic blood pressure; MDA, malondialdehyde; AOPP, advanced oxidation protein products; CRP, C-reactive protein; TC, total cholesterol; TG, triglycerides; HDL, high-density lipoprotein; LDL, low-density lipoprotein; TBARS, thiobarbituric acid reactive substance; Ca, total calcium; Ca++, calcium ionized. FBG, fasting blood glucose; HOMA-β, homeostasis model assessment of beta cell; TG, triglyceride; LDL, low-density lipoprotein; HDL, high-density lipoprotein; VLDL, very-low-density lipoprotein. CVD risk: cardiovascular risk HbA1c, hemoglobin A1c; 25(OH)D, 25-hydroxy vitamin D; HDL-C, high-density lipoprotein cholesterol; LDL-C, low-density lipoprotein cholesterol; PTH, parathyroid hormone; hsCRP, high-sensitivity C-reactive protein; pSBP, brachial systolic blood pressure; pDBP, brachial diastolic blood pressure; cSBP, central systolic blood pressure; Aix, radial augmentation index; baPWV, brachial–ankle pulse wave velocity; OA, daytime outdoor physical activity.

### 3.3. Risk of Bias Assessment in Individual Studies

**Figure 2:**
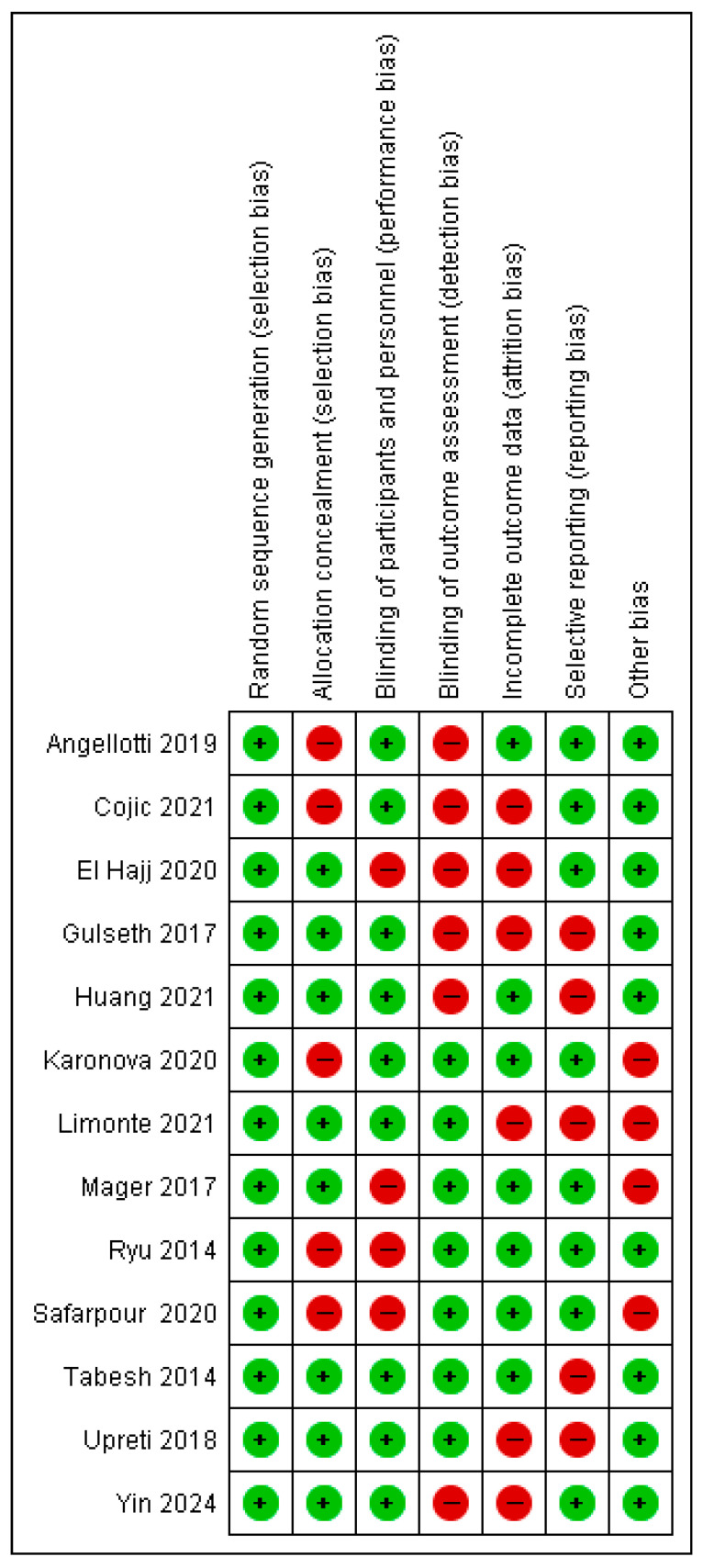
Overview of bias risk [17,20,23,28,34,38,40,42,43,44,45,49,50].

**Figure 3:**
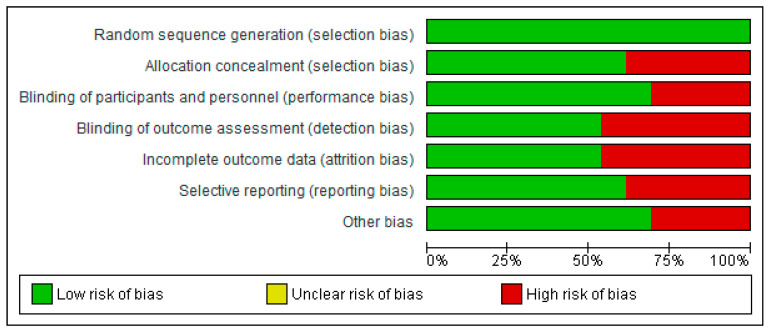
Diagram illustrating the risk of bias among the included studies.

The risk of bias assessment is illustrated in Figure 2 and Figure 3. For random sequence generation, all studies (100%) were rated as low risk [18,21,24,29,35,39,40,41,42,43,44,45,46].

- Allocation concealment: 61.5% of studies were assessed as low risk, while 38.5% were high risk [21,24,35,39,46].
- Blinding of participants and personnel: 69.27% were rated low risk, with 30.8% classified as high risk [39,40,45,46].
- Blinding of outcome assessment: 53.8% were low risk, and 46.26% were high risk [18,21,24,29,40,41].
- Incomplete outcome data: 53.8% were low risk, while 46.26% were high risk [21,29,40,41,42,44].
- Selective reporting: 61.57% of studies were classified as low risk, and 38.54% as high risk [18,41,42,43,44].

### 3.4. Synthesis of Results

The meta-analysis was performed using continuous variables in RevMan 5.4 (Cochrane Collaboration, London, UK). For each included study, group size (n), group means, and standard deviations (SDs) were entered. When SDs were not reported, they were estimated from confidence intervals using the formula:

SD = √(n) × (maximum range − minimum IQ range)/3.92.

The analysis applied the inverse variance method for continuous outcomes. A decrease in the measured parameter was interpreted as a positive effect, favoring the group with the largest reduction.

#### 3.4.1. Body Mass Index (BMI)

**Figure 4:**
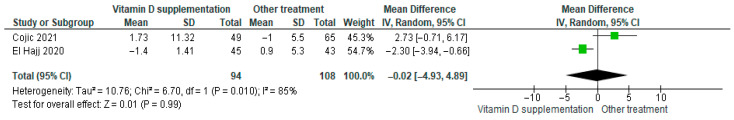
Forest plot for outcome BMI in patients treated with vitamin D versus other treatments [20,49].

Vitamin D supplementation was compared with placebo regarding its effect on BMI. The pooled analysis of two studies showed no significant difference between groups (MD = −0.02; 95% CI: −4.93 to 4.89; *p* = 0.99) (Figure 4) [21,40]. While the direction of the effect was consistent across studies, the confidence intervals overlapped. However, substantial heterogeneity was observed (*I*^2^ = 85%, *p* = 0.010). Based on the GRADE assessment, the certainty of this evidence was rated as very low (see Supplementary Table S4).

Although BMI decreased slightly in participants receiving vitamin D supplementation, the reduction was not statistically significant (*p* = 0.99).

#### 3.4.2. Vitamin D

**Figure 5:**
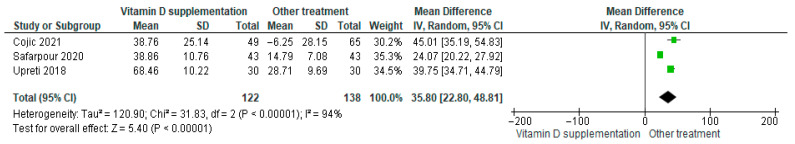
Forest plot for outcome vitamin D in patients treated with vitamin D versus other treatments [20,38,43].

Vitamin D supplementation was compared with placebo for its effect on serum vitamin D levels. The pooled analysis demonstrated a significant increase in vitamin D levels among participants receiving supplementation (MD = 35.80; 95% CI: 22.80 to 48.81; *p* < 0.0001) (Figure 5) [21,40]. The direction of the effect was consistent across studies, and confidence intervals overlapped. However, substantial heterogeneity was observed (*I*^2^ = 85%, *p* = 0.010). Based on the GRADE assessment, the certainty of this evidence was rated as very low (Supplementary Table S4).

Although vitamin D levels significantly improved following supplementation, the high heterogeneity and low certainty of evidence warrant cautious interpretation of these findings.

#### 3.4.3. Glycosylated Hemoglobin HbA1c%

**Figure 6:**
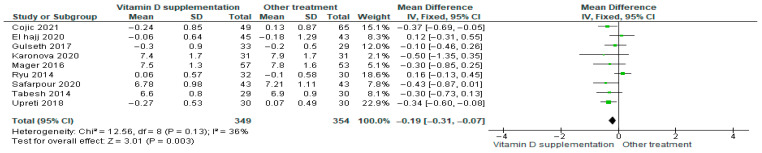
Forest plot for outcome glycosylated hemoglobin (HbA1c%) in patients treated with vitamin D versus other treatments [20,34,38,40,43,44,45,49,50].

Vitamin D supplementation was compared with placebo to evaluate its effect on glycosylated hemoglobin (HbA1c%). Pooled analysis from nine studies demonstrated a significant reduction in HbA1c among participants receiving vitamin D (MD = −0.19; 95% CI: −0.31 to −0.07; *p* = 0.003) (Figure 6) [21,35,39,40,41,43,44,45,46]. The direction of the effect was consistent across studies, with overlapping confidence intervals. Moderate heterogeneity was observed (*I*^2^ = 36%, *p* = 0.13). According to GRADE, the certainty of this evidence was rated as low (Supplementary Table S4).

These results indicate that vitamin D supplementation significantly reduces HbA1c%, reflecting an improvement in glycemic control that is clinically relevant for patients with diabetes.

#### 3.4.4. 25-Hydroxyvitamin D 25(OH)D

**Figure 7:**
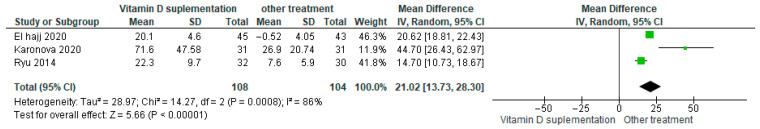
Forest plot for outcome 25-Hidroxivitamin D25(OH)D in patients treated with vitamin D versus other treatments [34,45,49].

Vitamin D supplementation was compared with placebo to evaluate its effect on glycosylated hemoglobin (HbA1c%). Pooled analysis from nine studies demonstrated a significant reduction in HbA1c among participants receiving vitamin D (MD = −0.19; 95% CI: −0.31 to −0.07; *p* = 0.003) (Figure 7) [21,35,39,40,41,43,44,45,46]. The direction of the effect was consistent across studies, with overlapping confidence intervals. Moderate heterogeneity was observed (*I*^2^ = 36%, *p* = 0.13). According to GRADE, the certainty of this evidence was rated as low (Supplementary Table S4).

These results indicate that vitamin D supplementation significantly reduces HbA1c%, reflecting an improvement in glycemic control that is clinically relevant for patients with diabetes.

#### 3.4.5. Homeostasis Model of Assessment Estimated Insulin Resistance HOMA-IR

**Figure 8:**
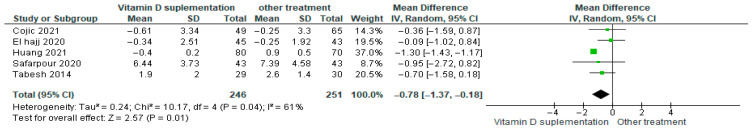
Forest plot for outcome HOMA-IR in patients treated with vitamin D versus other treatments according to Sin Huang [17,20,38,49,50].

Vitamin D supplementation was compared with placebo to evaluate its effect on insulin resistance measured by HOMA-IR. Pooled analysis from five studies demonstrated a significant reduction in HOMA-IR among participants receiving vitamin D (MD = −0.78; 95% CI: −1.37 to −1.18; *p* < 0.01) (Figure 8) [18,21,39,40,43]. The direction of the effect was consistent across studies, with overlapping confidence intervals. Moderate heterogeneity was observed (*I*^2^ = 61%, *p* = 0.04). Based on GRADE, the certainty of this evidence was rated as low (Supplementary Table S4).

These findings suggest that vitamin D supplementation may reduce insulin resistance in patients with diabetes, although the low certainty of evidence and observed heterogeneity warrant cautious interpretation.

#### 3.4.6. Homeostasis Model Assessment of Beta Cell Function (HOMA-β)

**Figure 9:**
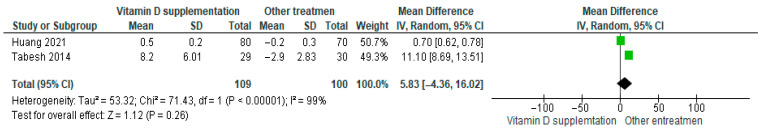
Forest plot for outcome HOMA-ꞵ in patients treated with vitamin D versus other treatments [17,50].

Vitamin D supplementation was compared with placebo to evaluate its effect on beta-cell function measured by HOMA-β. Pooled analysis from two studies showed no statistically significant difference between groups (MD = 5.83; 95% CI: −4.36 to 16.02; *p* = 0.26) (Figure 9) [18,43]. The direction of the effect was consistent, with overlapping confidence intervals. However, substantial heterogeneity was observed across studies (*I*^2^ = 99%, *p* < 0.00001). According to GRADE, the certainty of this evidence was rated as very low (Supplementary Table S4).

These results indicate that vitamin D supplementation does not produce a significant effect on beta-cell function in patients with diabetes.

#### 3.4.7. HDL-Cholesterol (mg/dL)

**Figure 10:**
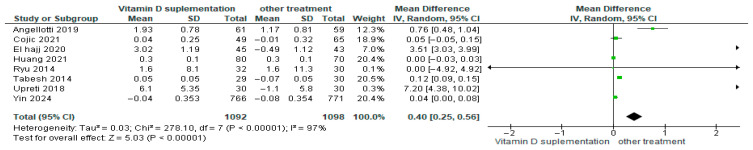
Forest plot for outcome HDL-cholesterol (mg/dL) in patients treated with vitamin D versus other treatments [17,20,23,28,43,45,49,50].

Vitamin D supplementation was compared with placebo to evaluate its effect on HDL-cholesterol levels. Pooled analysis from eight studies demonstrated a significant increase in HDL-cholesterol among participants receiving vitamin D (MD = 0.40; 95% CI: 0.25 to 0.56; *p* < 0.00001) (Figure 10) [18,21,24,29,40,43,44,46]. The direction of the effect was consistent, with overlapping confidence intervals across studies. However, substantial heterogeneity was observed (*I*^2^ = 97%, *p* < 0.00001). Based on GRADE, the certainty of this evidence was rated as very low (Supplementary Table S4).

These findings indicate that vitamin D supplementation significantly improves HDL-cholesterol compared to placebo in patients with diabetes despite the high heterogeneity and low certainty of the evidence.

#### 3.4.8. LDL-Cholesterol (mg/dL)

**Figure 11:**
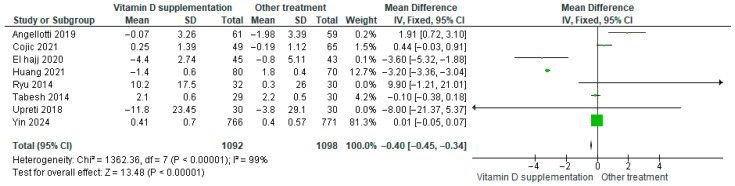
Forest plot for outcome LDL-cholesterol (mg/dL) in patients treated with vitamin D versus other treatments [17,20,23,28,43,45,49,50].

Vitamin D supplementation was compared with placebo to evaluate its effect on LDL-cholesterol levels. Pooled analysis from eight studies demonstrated a significant reduction in LDL among participants receiving vitamin D (MD = −0.40; 95% CI: −0.45 to −0.34; *p* < 0.00001) (Figure 11) [18,21,24,29,40,43,44,46]. The direction of the effect was consistent, with overlapping confidence intervals across studies. However, substantial heterogeneity was observed (*I*^2^ = 99%, *p* < 0.00001). According to GRADE, the certainty of this evidence was rated as low (Supplementary Table S4).

These results indicate that vitamin D supplementation significantly lowers LDL-cholesterol, which is clinically favorable for patients with diabetes.

#### 3.4.9. Parathyroid Hormone (PTH)

**Figure 12:**
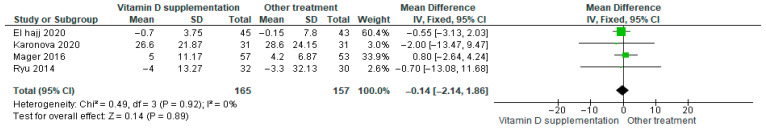
Forest plot for outcome PTH in patients treated with vitamin D versus other treatments [34,44,45,49].

Vitamin D supplementation was compared with placebo to assess its effect on parathyroid hormone (PTH) levels. Pooled analysis from four studies showed no significant difference between groups (MD = −0.14; 95% CI: −2.14 to 1.86; *p* = 0.89) (Figure 12) [35,40,45,46]. The direction of the effect was consistent, with overlapping confidence intervals across studies. Minimal heterogeneity was observed (*I*^2^ = 0%, *p* = 0.92). According to GRADE, the certainty of this evidence was rated as very low (Supplementary Table S4).

Although PTH levels tended to decrease slightly in participants receiving vitamin D supplementation, this change was not statistically significant (*p* = 0.89).

#### 3.4.10. Calcium (Ca^2+^)

**Figure 13:**
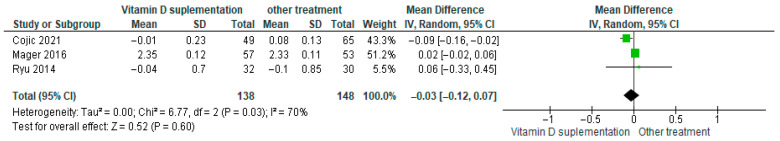
Forest plot for outcome calcium in patients treated with vitamin D versus other treatments [20,44,45].

Vitamin D supplementation was compared with placebo to evaluate its effect on serum calcium (Ca^2+^) levels. Pooled analysis from three studies showed no significant difference between groups (MD = −0.03; 95% CI: −0.12 to 0.07; *p* = 0.60) (Figure 13) [21,45,46]. The direction of the effect was consistent, with overlapping confidence intervals across studies. Moderate heterogeneity was observed (*I*^2^ = 70%, *p* = 0.03). According to GRADE, the certainty of this evidence was rated as very low (Supplementary Table S4).

Although calcium levels showed a slight decrease in the vitamin D supplementation group, this change was not statistically significant (*p* = 0.60).

#### 3.4.11. Total Cholesterol

**Figure 14:**
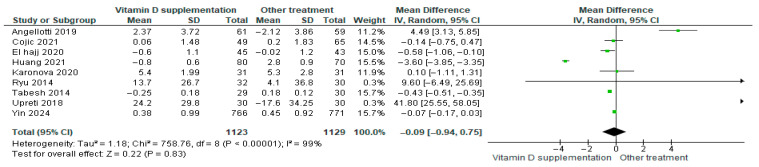
Forest plot for outcome total cholesterol in patients treated with vitamin D versus other treatments [17,20,23,28,34,43,45,49,50].

Vitamin D supplementation was compared with placebo to assess its effect on total cholesterol levels. Pooled analysis from nine studies showed a slight decrease in total cholesterol among participants receiving vitamin D (MD = −0.09; 95% CI: −0.94 to −0.75; *p* = 0.83) (Figure 14) [18,21,24,29,35,40,43,44,46]. The direction of the effect was consistent, with overlapping confidence intervals across studies. Substantial heterogeneity was observed (*I*^2^ = 99%, *p* < 0.00001). According to GRADE, the certainty of this evidence was rated as low (Supplementary Table S4).

Although total cholesterol decreased slightly in the vitamin D group, this change was not statistically significant (*p* = 0.83).

#### 3.4.12. Interleukin-6 (IL-6)

**Figure 15:**
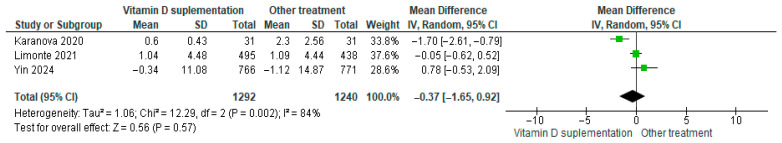
Forest plot for outcome IL-6 in patients treated with vitamin D versus other treatments [28,34,42].

Vitamin D supplementation was compared with placebo to evaluate its effect on interleukin-6 (IL-6) levels. Pooled analysis from three studies showed no significant difference between groups (MD = −0.37; 95% CI: −1.65 to 0.92; *p* = 0.57) (Figure 15) [29,35,42]. The direction of the effect was consistent, with overlapping confidence intervals across studies. Substantial heterogeneity was observed (*I*^2^ = 84%, *p* = 0.002). According to GRADE, the certainty of this evidence was rated as very low (Supplementary Table S4).

Although IL-6 levels tended to decrease slightly in participants receiving vitamin D supplementation, this change was not statistically significant (*p* = 0.57).

#### 3.4.13. Phosphorus

**Figure 16:**
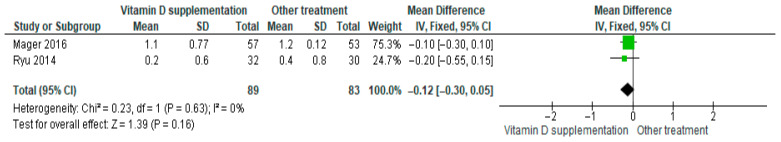
Forest plot for outcome phosphorus in patients treated with vitamin D versus other treatments [44,45].

Vitamin D supplementation was compared with placebo to evaluate its effect on serum phosphorus levels. Pooled analysis from two studies showed no significant difference between groups (MD = −0.12; 95% CI: −0.30 to 0.05; *p* = 0.16) (Figure 16) [45,46]. The direction of the effect was consistent, with overlapping confidence intervals. Minimal heterogeneity was observed across studies (*I*^2^ = 0%, *p* = 0.63). According to GRADE, the certainty of this evidence was rated as very low (Supplementary Table S4).

Although phosphorus levels tended to decrease slightly in participants receiving vitamin D supplementation, this change was not statistically significant (*p* = 0.16).

#### 3.4.14. Interleukin-1β (IL-1β)

**Figure 17:**
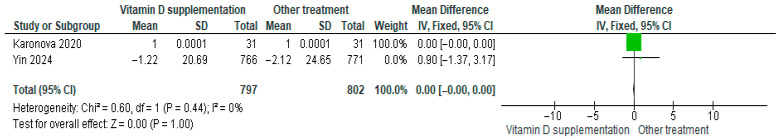
Forest plot for outcome IL-1B in patients treated with vitamin D versus other treatments [28,34].

Vitamin D supplementation was compared with placebo to evaluate its effect on interleukin-1β (IL-1β) levels. Pooled analysis from two studies showed no significant difference between groups (MD = −0.00; 95% CI: −0.00 to 0.00; *p* = 1.00) (Figure 17) [29,35]. The direction of the effect was consistent, with overlapping confidence intervals. Minimal heterogeneity was observed (*I*^2^ = 0%, *p* = 0.44). According to GRADE, the certainty of this evidence was rated as low (Supplementary Table S3).

These results indicate that IL-1β levels did not differ significantly between participants receiving vitamin D supplementation and those receiving placebo (*p* = 1.00).

#### 3.4.15. Fasting Insulin

**Figure 18:**
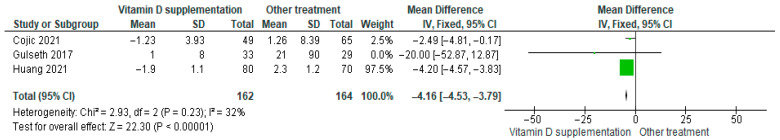
Forest plot for outcome fasting insulin in patients treated with vitamin D versus other treatments [17,20,40].

Vitamin D supplementation was compared with placebo to evaluate its effect on fasting insulin levels. Pooled analysis from three studies demonstrated a significant reduction in fasting insulin among participants receiving vitamin D (MD = −4.16; 95% CI: −4.53 to −3.79; *p* < 0.00001) (Figure 18) [18,21,41]. The direction of the effect was consistent, with overlapping confidence intervals. Moderate heterogeneity was observed (*I*^2^ = 32%, *p* = 0.23). According to GRADE, the certainty of this evidence was rated as very low (Supplementary Table S4).

These findings indicate that fasting insulin levels significantly decreased in the vitamin D supplementation group, representing a clinically favorable change for patients with diabetes (*p* < 0.00001).

#### 3.4.16. Triglycerides (TG)

**Figure 19:**
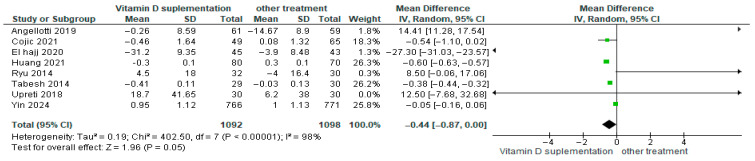
Forest plot for outcome triglycerides in patients treated with vitamin D versus other treatments [17,20,23,28,43,45,49,50].

Vitamin D supplementation was compared with placebo to assess its effect on triglyceride levels (TG). Pooled analysis from eight studies demonstrated a significant reduction in triglycerides among participants receiving vitamin D (MD = −0.44; 95% CI: −0.87 to 0.00; *p* = 0.05) (Figure 19) [18,21,24,29,40,43,44,46]. The direction of the effect was consistent, with overlapping confidence intervals across studies. Substantial heterogeneity was observed (*I*^2^ = 98%, *p* < 0.00001). According to GRADE, the certainty of this evidence was rated as very low (Supplementary Table S4).

These results indicate that triglyceride levels decreased significantly in the vitamin D supplementation group, representing a favorable effect for patients with diabetes (*p* = 0.05).

#### 3.4.17. C-Reactive Protein (CRP)

**Figure 20:**
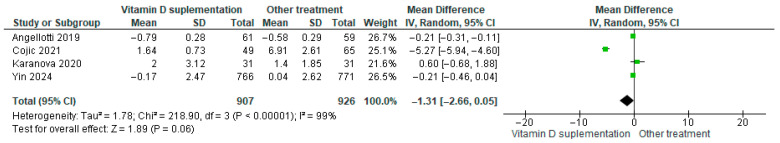
Forest plot for outcome C-reactive protein in patients treated with vitamin D versus other treatments [20,23,28,34].

Vitamin D supplementation was compared with placebo to assess its effect on C-reactive protein (CRP) levels. Pooled analysis from four studies indicated a decrease in CRP among participants receiving vitamin D (MD = −1.31; 95% CI: −2.66 to 0.05; *p* = 0.06) (Figure 20) [21,24,29,35]. The direction of the effect was consistent, with overlapping confidence intervals across studies. Substantial heterogeneity was observed (*I*^2^ = 99%, *p* < 0.00001). According to GRADE, the certainty of this evidence was rated as low (Supplementary Table S4).

Although CRP levels tended to decrease in the vitamin D supplementation group, this change was not statistically significant (*p* = 0.06).

#### 3.4.18. Fasting Plasma/Blood Glucose

**Figure 21:**
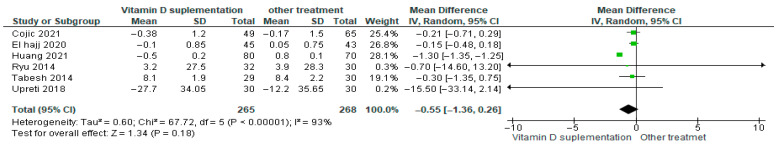
Forest plot for outcome fasting plasma/blood glucose in patients treated with vitamin D versus other treatments [17,20,43,45,49,50].

Vitamin D supplementation was compared with placebo to evaluate its effect on fasting plasma/blood glucose levels. Pooled analysis from six studies showed a reduction in fasting glucose among participants receiving vitamin D (MD = −0.55; 95% CI: −1.36 to 0.26; *p* = 0.18) (Figure 21) [18,21,40,43,44,46]. The direction of the effect was consistent, with overlapping confidence intervals across studies. Substantial heterogeneity was observed (*I*^2^ = 93%, *p* < 0.00001). According to GRADE, the certainty of this evidence was rated as very low (Supplementary Table S4).

Although fasting plasma glucose tended to decrease in the vitamin D supplementation group, this change was not statistically significant (*p* = 0.18).

#### 3.4.19. Systolic Blood Pressure (BP)

**Figure 22:**
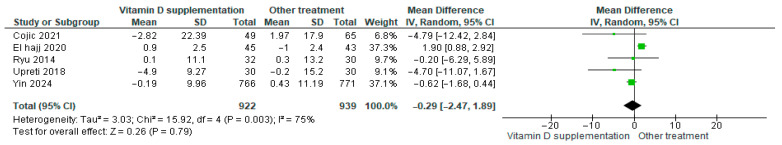
Forest plot for outcome systolic BP in patients treated with vitamin D versus another treatments [20,28,43,45,49].

Vitamin D supplementation was compared with placebo to evaluate its effect on systolic blood pressure. Pooled analysis from five studies showed no significant difference between groups (b) (Figure 22) [21,29,40,44,46]. The direction of the effect was consistent, with overlapping confidence intervals across studies, although substantial heterogeneity was observed (*I*^2^ = 75%, *p* = 0.003). According to GRADE, the certainty of this evidence was rated as very low (Supplementary Table S4).

While systolic blood pressure tended to decrease slightly in the vitamin D group, this change was not statistically significant (*p* = 0.79).

#### 3.4.20. Diastolic Blood Pressure (BP)

**Figure 23:**
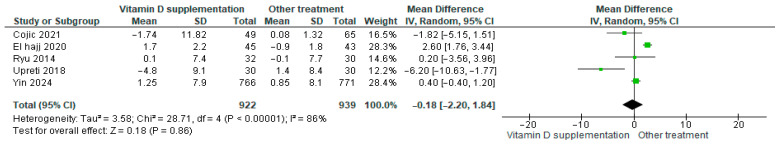
Forest plot for outcome diastolic BP in patients treated with vitamin D versus other treatments [20,28,43,45,49].

Vitamin D supplementation was compared with placebo to evaluate its effect on diastolic blood pressure. Pooled analysis from five studies indicated no significant difference between groups (MD = −0.18; 95% CI: −2.20 to 1.84; *p* = 0.86) (Figure 23) [21,29,40,44,46]. The direction of the effect was consistent, with overlapping confidence intervals across studies. Substantial heterogeneity was observed (*I*^2^ = 86%, *p* < 0.00001). According to GRADE, the certainty of this evidence was rated as low (Supplementary Table S4).

Although diastolic blood pressure tended to decrease slightly in the placebo group, this change was not statistically significant (*p* = 0.86).

#### 3.4.21. QUIKI (Quantitative Insulin Sensitivity Check Index)

**Figure 24:**
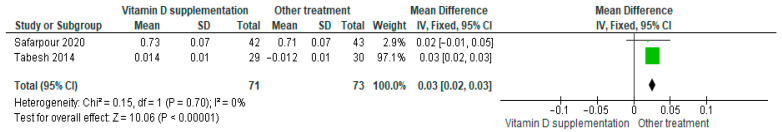
Forest plot for outcome QUIKI (Quantitative Insulin Sensitivity Check Index) in patients treated with vitamin D versus another treatments [38,50].

Vitamin D supplementation was compared with placebo to evaluate its effect on insulin sensitivity using the QUICKI index. Pooled analysis from two studies demonstrated a significant improvement in insulin sensitivity among participants receiving vitamin D (MD = 0.03; 95% CI: 0.02 to 0.03; *p* < 0.00001) (Figure 24) [39,43]. The direction of the effect was consistent, with overlapping confidence intervals. Minimal heterogeneity was observed across studies (*I*^2^ = 0%, *p* = 0.70). According to GRADE, the certainty of this evidence was rated as low (Supplementary Table S4).

These results indicate that the QUICKI scores significantly improved in the vitamin D supplementation group compared to placebo, reflecting enhanced insulin sensitivity (*p* < 0.00001).

### 3.5. Adverse Effects of Vitamin D

Although vitamin D is essential for numerous physiological processes, it can also cause a range of adverse effects, from mild to severe. The most frequently reported side effects include nausea, vomiting from high-dose intake, abdominal discomfort, diarrhea, and general fatigue. While these effects are generally not severe enough to compromise hemodynamic stability, if left unaddressed, they can cause significant discomfort and potentially progress to a more symptomatic state. More serious complications include hypercalcemia, which occurs due to increased intestinal calcium absorption and simultaneous mobilization of calcium from bones, potentially elevating the risk of osteoporosis. Excess vitamin D intake can also lead to nephrocalcinosis and, if untreated, may progress to kidney failure. Additionally, high doses may result in cardiac arrhythmias, including monomorphic or polymorphic ventricular tachycardia and atrial fibrillation. When initiating supplementation, it is important to consider potential interactions with other medications. Careful monitoring of serum vitamin D levels and the patient’s overall health is critical, as proper supervision can prevent serious complications [13,15,18,21,25,27].

### 3.6. Sensitivity Analysis

An exclusion analysis was conducted to assess the impact of individual studies on the results. For HOMA-IR, removing the study by Huang et al., 2021 [17] led to changes in statistical significance, indicating that this study influenced the outcome (Supplementary Figure S1). For PTH, exclusion of El Hajj et al., 2021 [49] had no impact, as the statistical results remained nonsignificant in both analyses (Supplementary Figure S2). For fasting insulin, removing the study by Mager et al., 2016 [44] revealed statistically significant differences favoring the vitamin D supplementation group; including this study eliminated the difference, indicating that it substantially influenced the outcome (Supplementary Figure S3).

### 3.7. Meta-Regression

Meta-regression analyses were conducted using moderators including age, vitamin D dose (mg), frequency of omega-3 supplementation per week, and total duration of vitamin D supplementation. Statistically significant associations were observed for several outcomes. For 25(OH)D levels, both dose (β = −0.02, 95% CI −0.02 to −0.01, *p* = 0.0016) and treatment duration in weeks (β = 0.01, 95% CI 0.0001 to 0.01, *p* = 0.036) were significant. For HOMA-IR, dose frequency per week was significant (β = −0.56, 95% CI −0.94 to −0.19, *p* = 0.0033). Vitamin D levels were significantly affected by total treatment duration in weeks (β = −0.61, 95% CI −0.71 to −0.52, *p* < 0.00001). For C-reactive protein, the dose in mg was significant (β = −0.61, 95% CI −0.71 to −0.51, *p* < 0.0001). For fasting plasma glucose, three moderators showed significance: subject age (β = 0.03, 95% CI 0.03 to 0.04, *p* < 0.0001), dose in mg (β = −0.08, 95% CI −0.15 to −0.0001, *p* = 0.038), and weekly dose frequency (β = −0.1, 95% CI −0.15 to −0.06, *p* < 0.0001) (Table 2).

## 4. Discussion

In this study, vitamin D supplementation was associated with significant improvements in several metabolic parameters. Notably, reductions were observed in BMI, an indicator of insulin resistance and diabetes; glycosylated hemoglobin (HbA1c%), which reflects average blood glucose levels over three months; HOMA-IR, a marker of insulin resistance; LDL cholesterol; total cholesterol; and triglycerides. These effects are consistent with vitamin D’s role in decreasing triglyceride synthesis, increasing HDL cholesterol, and reducing total cholesterol. Additionally, a reduction in IL-6, an inflammatory cytokine elevated in patients with excess visceral adiposity, was observed. This effect may be mediated through inhibition of the NF-κB pathway by vitamin D, contributing to reduced systemic inflammation. Collectively, the decrease in these parameters suggests a beneficial relationship between vitamin D supplementation, improved metabolic outcomes, and potentially reduced morbidity in patients with diabetes [50,51,52,53,54,55].

Fasting insulin, C-reactive protein, and fasting glucose—typically elevated in diabetes—also showed decreases, likely reflecting vitamin D’s protective effects on pancreatic β cells. Minor reductions were observed for phosphorus, parathyroid hormone (PTH), and calcium (Ca^2+^).

In contrast, the placebo or non-vitamin D groups experienced declines in 25-hydroxyvitamin D levels, HOMA-β (a measure of β cell activity), HDL cholesterol, systolic and diastolic blood pressure, mean arterial pressure (MAP), and the QUIKI index. These changes underscore the importance of supplementation in maintaining metabolic homeostasis.

Previous reviews provide additional context. Hartweg (2009) [56] investigated marine-derived omega-3 PUFAs in type 2 diabetes (T2D), showing reductions in triglycerides and improvements in thrombogenesis, though the LDL levels increased. Gu et al. (2017) [36] found that vitamin D supplementation in T2D patients with deficiency significantly reduced HbA1c and fasting blood glucose. Huang et al. (2013) [26] reported improvements in 25(OH)D levels and insulin resistance with oral vitamin D supplementation but no effect on fasting glucose, insulin, or HbA1c. Chou et al. (2021) [18] observed reductions in CRP but no changes in TNF-α or IL-6. Barbarawi et al. (2020) [57] found that medium-to-high doses of vitamin D reduced progression to T2D in prediabetic individuals. Chewcharat et al. (2020) [58] reported positive effects of omega-3 on proteinuria without adverse effects on HbA1c, total cholesterol, or LDL cholesterol. Putranto et al. (2022) [59] showed vitamin D supplementation improved depressive symptoms in T2D patients.

Our findings align with these results: vitamin D supplementation decreased HbA1c, reflecting improved glycemic control, likely through direct effects on pancreatic β cells and modulation of intracellular calcium, which is critical for insulin secretion [60].

Vitamin D has diverse extraskeletal effects. Its hormonal system, derived from 7-dehydrocholesterol, regulates bone and mineral metabolism, immune function, inflammation, blood pressure, and cellular proliferation and differentiation. Vitamin D enhances insulin secretion from pancreatic β cells, improves peripheral insulin sensitivity, and exerts anti-inflammatory and immunomodulatory effects. Both cholecalciferol (D3) and ergocalciferol (D2) are converted to the active form, 1,25-dihydroxyvitamin D, in the liver, kidneys, pancreas, and immune cells. Active vitamin D helps regulate calcium/phosphorus balance, inflammation, insulin resistance, and obesity.

In individuals with T1D and T2D, 1,25(OH)2D interacts with β cell receptors, modulates the renin–angiotensin system, enhances insulin secretion via calcium channels, and increases insulin sensitivity through insulin receptor expression and PPAR-δ activation. Vitamin D also mitigates chronic inflammation by downregulating proinflammatory cytokines and promoting calbindin release, which protects against β cell apoptosis. Consequently, fasting plasma glucose decreased in patients receiving vitamin D supplementation. Supporting evidence includes the study of Holick et al. (2007) [61], which demonstrated improved insulin sensitivity with adequate vitamin D, and recent research in Chilean children, indicating improved insulin synthesis and glucose control with supplementation.

High BMI is frequently associated with low serum 25(OH)D levels, potentially due to sequestration in adipose tissue [55]. Low vitamin D correlates with elevated waist circumference, hypertriglyceridemia, and high LDL levels [45]. Supplementation reduces LDL, triglycerides, and total cholesterol, supporting benefits in obesity. Vitamin D receptors are expressed in visceral and subcutaneous adipose tissue, highlighting its role in fat metabolism [62].

Vitamin D also regulates immune function, influencing monocytes, macrophages, dendritic cells, T cells, and B cells. It modulates genes involved in cell proliferation, differentiation, and apoptosis, potentially preventing β cell destruction and improving insulin production [56]. Clinical and experimental studies indicate that vitamin D supports β cell morphology, insulin production, and receptor expression while modulating inflammatory responses. These mechanisms underline vitamin D’s role in diabetes management and prevention, as reductions in HbA1c correlate with lower risks of microvascular and macrovascular complications.

## 5. Study Limitations

This review has several limitations. Publication and authorship biases may have led to the omission of relevant studies. Search strategies may have affected study inclusion due to sensitivity and specificity limitations. Some studies were excluded because of inconsistent units, unclear dosing regimens (daily vs. weekly), or undefined follow-up durations, introducing potential reporting bias. Additionally, variability in supplementation doses, study populations, and outcome measures required grouping of values, which limited precision and may have influenced observed effects. This heterogeneity introduces bias and complicates standardization of dosing recommendations.

## 6. Conclusions

Vitamin D supplementation positively affects multiple metabolic and inflammatory parameters in patients with diabetes and prediabetes. Supplementation was associated with significant reductions in HbA1c, HOMA-IR, HOMA-β, LDL cholesterol, total cholesterol, triglycerides, fasting insulin, fasting plasma glucose, and C-reactive protein, and it increased the likelihood of reversion to normoglycemia in prediabetic individuals. These findings support vitamin D’s role in modulating insulin secretion, insulin sensitivity, and systemic inflammation. Our meta-regression highlights that treatment dose and duration are key factors influencing outcomes, emphasizing the need for primary studies to define optimal dosing and supplementation periods. Although variability may be influenced by sun exposure, geographic location, genetics, or diet, the inclusion of high-quality trials and combined quantitative and qualitative analyses reinforces the robustness of our conclusions. Large, well-designed randomized trials are warranted to confirm these findings and establish optimal dosing strategies for specific populations.

## Figures and Tables

**Table 2:** Meta Regression.

Outcome	Moderator	B	95% LLCI	95% ULCI	*p*-Value
HDL	Age	0.04	−0.08	0.16	0.48384
HDL	Dose (mg)	−0.15	−0.47	0.18	0.38439
HDL	Treatment weeks	0.04	−0.09	0.17	0.56768
HDL	Dose frequency per week	−0.27	−0.59	0.06	0.10899
LDL	Age	0.04	−0.08	0.16	0.48384
LDL	Dose (mg)	−0.15	−0.47	0.18	0.38439
LDL	Treatment weeks	0.04	−0.09	0.17	0.56768
LDL	Dose frequency per week	−0.27	−0.59	0.06	0.10899
Cholesterol	Age	0.13	−0.05	0.31	0.15484
Cholesterol	Dose (mg)	−0.34	−0.7	0.03	0.07351
Cholesterol	Treatment weeks	0.13	−0.04	0.31	0.14254
Cholesterol	Dose frequency per week	−0.24	−0.83	0.35	0.42467
Triglycerides	Age	−0.19	−0.91	0.53	0.60387
Triglycerides	Dose (mg)	−0.09	−2.09	1.9	0.92790
Triglycerides	Treatment weeks	0.29	−0.43	1.01	0.43522
Triglycerides	Dose frequency per week	0.67	−1.29	2.64	0.50090
25(OH)D	Age	−0.01	−0.03	0.02	0.70183
25(OH)D	Dose (mg)	−0.02	−0.02	−0.01	0.00166
25(OH)D	Treatment weeks	0.01	0	0.01	0.00321
25(OH)D	Dose frequency per week	0.01	−0.02	0.03	0.66830
HOMA-IR	Age	0.41	−0.01	0.83	0.05565
HOMA-IR	Dose (mg)	−0.21	−0.72	0.29	0.40873
HOMA-IR	Treatment weeks	0.01	−0.14	0.16	0.90321
HOMA-IR	Dose frequency per week	−0.56	−0.94	−0.19	0.00335
Calcium	Age	0.01	−0.01	0.02	0.42613
Calcium	Dose (mg)	−0.01	−0.03	0.01	0.31830
Calcium	Treatment weeks	0	0	0.01	0.31830
Calcium	Dose frequency per week	0	−0.03	0.02	0.76253
Diastolic BP	Age	0.03	−0.19	0.26	0.78868
Diastolic BP	Dose (mg)	−0.25	−1.16	0.67	0.59782
Diastolic BP	Treatment weeks	0	−0.4	0.4	0.99069
Diastolic BP	Dose frequency per week	0.19	−0.54	0.91	0.61071
Systolic BP	Age	0.04	−0.14	0.22	0.69207
Systolic BP	Dose (mg)	−0.6	−1.65	0.46	0.26602
Systolic BP	Treatment weeks	0.1	−0.23	0.43	0.54804
Systolic BP	Dose frequency per week	−0.01	−0.71	0.7	0.98728
IL-6	Age	−0.03	−0.14	0.08	0.56078
IL-6	Dose (mg)	−2.02	−4.51	0.47	0.11225
IL-6	Dose frequency per week	0.27	−0.23	0.77	0.29067
Level VD	Age	0.96	−1.4	3.33	0.42301
Level VD	Dose (mg)	1.73	−2.17	5.62	0.38471
Level VD	Treatment weeks	1.05	0.68	1.42	0.00000
Level VD	Dose frequency per week	2.2	−2.47	6.87	0.35614
PTH	Age	0.11	−0.74	0.97	0.79645
PTH	Dose (mg)	−3.3	−13.18	6.58	0.51246
PTH	Dose frequency per week	0.2	−0.61	1	0.62987
C-reactive Protein	Age	−0.07	−0.29	0.15	0.54776
C-reactive Protein	Dose (mg)	−0.61	−0.71	−0.52	0.00000
C-reactive Protein	Treatment weeks	0.07	−0.11	0.24	0.44845
C-reactive Protein	Dose frequency per week	−0.41	−1.53	0.7	0.46732
Fasting Insulin	Age	0.05	−0.21	0.3	0.72676
Fasting Insulin	Dose (mg)	−0.28	−1.68	1.13	0.69917
Fasting Insulin	Treatment weeks	0	−0.66	0.66	0.99293
Fasting Insulin	Dose frequency per week	0.07	−1.34	1.47	0.92545
Fasting plasma blood glucose	Age	0.03	0.03	0.04	0.00000
Fasting plasma blood glucose	Dose (mg)	−0.08	−0.15	0	0.03875
Fasting plasma blood glucose	Treatment weeks	−0.03	−0.12	0.05	0.42847
Fasting plasma blood glucose	Dose frequency per week	−0.1	−0.15	−0.06	0.00000
Glycosylated hemoglobin	Age	0.01	−0.02	0.04	0.39986
Glycosylated hemoglobin	Dose (mg)	−0.01	−0.06	0.04	0.68259
Glycosylated hemoglobin	Treatment weeks	0.01	−0.01	0.03	0.41729
Glycosylated hemoglobin	Dose frequency per week	0.03	0	0.06	0.05730

## Supplementary Materials

The following supporting information can be downloaded at: https://www.mdpi.com/article/10.3390/nu17182991/s1, Figure S1: Forest plot Forest plot for outcome HOMA IR without author Huang et al., 2021 [17]; Figure S2: Forest plot for outcome PTH without author El Hajj et al., 2020 [49]; Figure S3: Forest plot for outcome Calcium, without author Mager et al., 2016 [44]; Table S1: PRISMA; Table S2: Details of the search strategy; Table S3: Excluded studies and the reasons for their exclusion; Table S4: Summary of Findings (SoF) and quality of evidence (GRADE) for vitamin D treatment in patients with diabetes mellitus.
