# Supplementary material for: Effectiveness of Vitamin D Supplementation on Biochemical, Clinical, and Inflammatory Parameters in Patients with Different Types of Diabetes: A Systematic Review and Meta-Analysis
Source: Nutrients. 2025 Sep 18;17(18):2991. doi: 10.3390/nu17182991 (PMC12472623; doi:10.3390/nu17182991)
Supplement: Supplementary file 1 [file nutrients-17-02991-s001.zip › nutrients-3820502-supplementary.pdf]

Supplementary Tables

Table S1. PRISMA.

Section and Topic	Item #	Checklist item	Location where item is reported
**TITLE**			
Title	1	Identify the report as a systematic review.	Title page
**ABSTRACT**			
Abstract	2	See the PRISMA 2020 for Abstracts checklist.	Yes
**INTRODUCTION**			
Rationale	3	Describe the rationale for the review in the context of existing knowledge.	Pages 2–3
Objectives	4	Provide an explicit statement of the objective(s) or question(s) the review addresses.	Pages 4
**METHODS**			
Eligibility criteria	5	Specify the inclusion and exclusion criteria for the review and how studies were grouped for the syntheses.	Pages 4
Information sources	6	Specify all databases, registers, websites, organisations, reference lists and other sources searched or consulted to identify studies. Specify the date when each source was last searched or consulted.	Page 5
Search strategy	7	Present the full search strategies for all databases, registers and websites, including any filters and limits used.	Page 5
Selection process	8	Specify the methods used to decide whether a study met the inclusion criteria of the review, including how many reviewers screened each record and each report retrieved, whether they worked independently, and if applicable, details of automation tools used in the process.	Page 4–5
Data collection process	9	Specify the methods used to collect data from reports, including how many reviewers collected data from each report, whether they worked independently, any processes for obtaining or confirming data from study investigators, and if applicable, details of automation tools used in the process.	Pages 6
Data items	10a	List and define all outcomes for which data were sought. Specify whether all results that were compatible with each outcome domain in each study were sought (e.g., for all measures, time points, analyses), and if not, the methods used to decide which results to collect.	Pages 6
	10b	List and define all other variables for which data were sought (e.g., participant and intervention characteristics, funding sources). Describe any assumptions made about any missing or unclear information.	Pages 6
Study risk of bias assessment	11	Specify the methods used to assess risk of bias in the included studies, including details of the tool(s) used, how many reviewers assessed each study and whether they worked independently, and if applicable, details of automation tools used in the process.	Page 6
Effect measures	12	Specify for each outcome the effect measure(s) (e.g., risk ratio, mean difference) used in the synthesis or presentation of results.	Pages 6
Synthesis methods	13a	Describe the processes used to decide which studies were eligible for each synthesis (e.g., tabulating the study intervention characteristics and comparing against the planned groups for each synthesis (item #5)).	Page 6
	13b	Describe any methods required to prepare the data for presentation or synthesis, such as handling of missing summary statistics, or data conversions.	Pages 6

	13c	Describe any methods used to tabulate or visually display results of individual studies and syntheses.	Pages 6
	13d	Describe any methods used to synthesize results and provide a rationale for the choice(s). If meta-analysis was performed, describe the model(s), method(s) to identify the presence and extent of statistical heterogeneity, and software package(s) used.	Pages 6
	13e	Describe any methods used to explore possible causes of heterogeneity among study results (e.g., subgroup analysis, meta-regression).	Pages 7
	13f	Describe any sensitivity analyses conducted to assess robustness of the synthesized results.	Pages 7–8
Reporting bias assessment	14	Describe any methods used to assess risk of bias due to missing results in a synthesis (arising from reporting biases).	Page 7–8
Certainty assessment	15	Describe any methods used to assess certainty (or confidence) in the body of evidence for an outcome.	Page 7–8
**RESULTS**			
Study selection	16a	Describe the results of the search and selection process, from the number of records identified in the search to the number of studies included in the review, ideally using a flow diagram.	Page 9
	16b	Cite studies that might appear to meet the inclusion criteria, but which were excluded, and explain why they were excluded.	Page 9
Study characteristics	17	Cite each included study and present its characteristics.	Page 10
Risk of bias in studies	18	Present assessments of risk of bias for each included study.	Page 11
Results of individual studies	19	For all outcomes, present, for each study: (a) summary statistics for each group (where appropriate) and (b) an effect estimate and its precision (e.g., confidence/credible interval), ideally using structured tables or plots.	Supplemental Table 1
Results of syntheses	20a	For each synthesis, briefly summarise the characteristics and risk of bias among contributing studies.	Page 11
	20b	Present results of all statistical syntheses conducted. If meta-analysis was done, present for each the summary estimate and its precision (e.g., confidence/credible interval) and measures of statistical heterogeneity. If comparing groups, describe the direction of the effect.	Supplemental tables 1 and 3
	20c	Present results of all investigations of possible causes of heterogeneity among study results.	Supplemental figures 3 to 8
	20d	Present results of all sensitivity analyses conducted to assess the robustness of the synthesized results.	Supplemental figures 3 to 8
Reporting biases	21	Present assessments of risk of bias due to missing results (arising from reporting biases) for each synthesis assessed.	Supplemental Table 3
Certainty of evidence	22	Present assessments of certainty (or confidence) in the body of evidence for each outcome assessed.	Supplemental figures 3 to 8
**DISCUSSION**			
Discussion	23a	Provide a general interpretation of the results in the context of other evidence.	Pages 10 to 14
	23b	Discuss any limitations of the evidence included in the review.	Pages 11 and 12
	23c	Discuss any limitations of the review processes used.	Pages 12 and 13
	23d	Discuss implications of the results for practice, policy, and future research.	Pages 12 to 14

**OTHER INFORMATION**			
Registration and protocol	24a	Provide registration information for the review, including register name and registration number, or state that the review was not registered.	CRD420250655371.
	24b	Indicate where the review protocol can be accessed, or state that a protocol was not prepared.	N/A
	24c	Describe and explain any amendments to information provided at registration or in the protocol.	N/A
Support	25	Describe sources of financial or non-financial support for the review, and the role of the funders or sponsors in the review.	Page 15
Competing interests	26	Declare any competing interests of review authors.	Page 15
Availability of data, code and other materials	27	Report which of the following are publicly available and where they can be found: template data collection forms; data extracted from included studies; data used for all analyses; analytic code; any other materials used in the review.	Methods, 2.3 Electronic search

From: Page MJ, McKenzie JE, Bossuyt PM, Boutron I, Hoffmann TC, Mulrow CD, et al. The PRISMA 2020 statement: an updated guideline for reporting systematic reviews. BMJ 2021;372:n71. doi: 10.1136/bmj.n71. For more information, visit: http://www.prisma-statement.org.

Table S2. Details of the search strategy.

Database	Search Strategy	Results	
		20-03-25	04-08-2025
Medline	Search: **vitamin D supplementation OR Vitamin d OR Vitamin D supplement OR Vitamin D supplements AND diabete OR impaired glucose toleranc NOT review NOT animals** Filters: **Randomized Controlled Trial** (((((((“vitamin d”[Supplementary Concept] OR “vitamin d”[All Fields] OR “ergocalciferols”[Supplementary Concept] OR “ergocalciferols”[All Fields] OR “vitamin d”[MeSH Terms] OR “ergocalciferols”[MeSH Terms]) AND (“supplemental”[All Fields] OR “supplementating”[All Fields] OR “supplementation”[All Fields] OR “supplementation s”[All Fields] OR “supplementations”[All Fields] OR “supplemention”[All Fields])) OR (“vitamin d”[Supplementary Concept] OR “vitamin d”[All Fields] OR “ergocalciferols”[Supplementary Concept] OR “ergocalciferols”[All Fields] OR “vitamin d”[MeSH Terms] OR “ergocalciferols”[MeSH Terms]) OR ((“vitamin d”[Supplementary Concept] OR “vitamin d”[All Fields] OR “ergocalciferols”[Supplementary Concept] OR “ergocalciferols”[All Fields] OR “vitamin d”[MeSH Terms] OR “ergocalciferols”[MeSH Terms]) AND (“dietary	548	548

	supplements"[MeSH Terms] OR ("dietary"[All Fields] AND "supplements"[All Fields]) OR "dietary supplements"[All Fields] OR "supplement"[All Fields] OR "supplement s"[All Fields] OR "supplemented"[All Fields] OR "supplementing"[All Fields] OR "supplements"[All Fields])) OR (("vitamin d"[Supplementary Concept] OR "vitamin d"[All Fields] OR "ergocalciferols"[Supplementary Concept] OR "ergocalciferols"[All Fields] OR "vitamin d"[MeSH Terms] OR "ergocalciferols"[MeSH Terms]) AND ("dietary supplements"[MeSH Terms] OR ("dietary"[All Fields] AND "supplements"[All Fields]) OR "dietary supplements"[All Fields] OR "supplement"[All Fields] OR "supplement s"[All Fields] OR "supplemented"[All Fields] OR "supplementing"[All Fields] OR "supplements"[All Fields]))) AND ("diabete"[All Fields] OR "diabetes mellitus"[MeSH Terms] OR ("diabetes"[All Fields] AND "mellitus"[All Fields]) OR "diabetes mellitus"[All Fields] OR "diabetes"[All Fields] OR "diabetes insipidus"[MeSH Terms] OR ("diabetes"[All Fields] AND "insipidus"[All Fields]) OR "diabetes insipidus"[All Fields] OR "diabetic"[All Fields] OR "diabetics"[All Fields] OR "diabets"[All Fields])) OR (("impair"[All Fields] OR "impaired"[All Fields] OR "impairement"[All Fields] OR "impairements"[All Fields] OR "impairing"[All Fields] OR "impairment"[All Fields] OR "impairments"[All Fields] OR "impairs"[All Fields]) AND ("glucose"[Supplementary Concept] OR "glucose"[All Fields] OR "glucose"[MeSH Terms] OR "glucoses"[All Fields] OR "glucose s"[All Fields]) AND "toleranc"[All Fields])) NOT ("review"[Publication Type] OR "review literature as topic"[MeSH Terms] OR "review"[All Fields])) NOT ("animals"[MeSH Terms:noexp] OR "animals"[All Fields])) AND (randomizedcontrolledtrial[Filter])		
Wos	**vitamin D supplementation OR Vitamin d OR Vitamin D supplement OR Vitamin D supplements AND diabete OR impaired glucose toleranc NOT review NOT animals** Filters: **Randomized Controlled Trial**	90	90
CINAHL	**vitamin D supplementation OR Vitamin d OR Vitamin D supplement OR Vitamin D supplements AND diabete OR impaired glucose**	11	12

	**toleranc NOT review NOT animals** Filters: **Randomized Controlled Trial**		
SCOPUS	**vitamin D supplementation OR Vitamin d OR Vitamin D supplement OR Vitamin D supplements AND diabete OR impaired glucose toleranc NOT review NOT animals** Filters: **Randomized Controlled Trial**	111	113
Google Scholar	**vitamin D supplementation OR Vitamin d OR Vitamin D supplement OR Vitamin D supplements AND diabete OR impaired glucose toleranc NOT review NOT animals** Filters: **Randomized Controlled Trial**	191	193
	Total	851	856

* All searches were carried out on 4 September 2025.

Table S3. Excluded studies and the reasons for their exclusion.

N°	Reference	Reason
1	de Boer IH, Zelnick LR, Ruzinski J, Friedenberg G, Duszlak J, Bubes VY, Hoofnagle AN, Thadhani R, Glynn RJ, Buring JE, Sesso HD, Manson JE. Effect of Vitamin D and Omega-3 Fatty Acid Supplementation on Kidney Function in Patients With Type 2 Diabetes: A Randomized Clinical Trial. JAMA. 2019 Nov 19;322(19):1899-1909. doi: 10.1001/jama.2019.17380. Erratum in: JAMA. 2020 Jul 7;324(1):103. doi: 10.1001/jama.2020.10205. PMID: 31703120; PMCID: PMC6865245.	Effect on the kidney
2	Javed Z, Papageorgiou M, Deshmukh H, Kilpatrick ES, Mann V, Corless L, Abouda G, Rigby AS, Atkin SL, Sathyapalan T. A Randomized, Controlled Trial of Vitamin D Supplementation on Cardiovascular Risk Factors, Hormones, and Liver Markers in Women with Polycystic Ovary Syndrome. Nutrients. 2019 Jan 17;11(1):188. doi: 10.3390/nu11010188. PMID: 30658483; PMCID: PMC6356309.	Vitamin D supplementation is used to assess hormonal changes in women with polycystic ovary syndrome.
3	Al-Daghri NM, Alkharfy KM, Khan N, Alfawaz HA, Al-Ajlan AS, Yakout SM, Alokail MS. Vitamin D supplementation and serum levels of magnesium and selenium in type 2 diabetes mellitus patients: gender dimorphic changes. Int J Vitam Nutr Res. 2014;84(1-2):27-34. doi: 10.1024/0300-9831/a000190. PMID: 25835233.	They measure magnesium and selenium levels.
4	Byrn MA, Adams W, Penckofer S, Emanuele MA. Vitamin D Supplementation and Cognition in People with Type 2 Diabetes: A Randomized Control Trial. J Diabetes Res. 2019 Oct 30;2019:5696391. doi: 10.1155/2019/5696391. PMID: 31781666; PMCID: PMC6875298.	Assess cognitive function.
5	Khadilkar A, Oza C, Antani M, Shah N, Lohiya N, Khadilkar V, Bhor S, Kajale N, Gondhalekar K, More C, Katapally TR, Mughal Z, Bhawra J, Padidela R. Effect of Calcium and Vitamin D Supplementation (Dairy vs. Pharmacological) on Bone Health of Underprivileged Indian Children and Youth with Type-1 Diabetes: A Randomized Controlled Trial. J Clin Densitom. 2024 Apr-Jun;27(2):101468. doi: 10.1016/j.jocd.2024.101468. Epub 2024 Jan 26. PMID: 38325238.	Bone health assessed in children.
6	Kota SK, Jammula S, Kota SK, Tripathy PR, Panda S, Modi KD. Effect of vitamin D supplementation in type 2 diabetes patients with pulmonary tuberculosis. Diabetes	They focus on patients with

	Metab Syndr. 2011 Apr-Jun;5(2):85-9. doi: 10.1016/j.dsx.2012.02.021. Epub 2012 Mar 15. PMID: 22813409.	pulmonary tuberculosis.
7	Omidian M, Mahmoudi M, Abshirini M, Eshraghian MR, Javanbakht MH, Zarei M, Hasani H, Djalali M. Effects of vitamin D supplementation on depressive symptoms in type 2 diabetes mellitus patients: Randomized placebo-controlled double-blind clinical trial. Diabetes Metab Syndr. 2019 Jul-Aug;13(4):2375-2380. doi: 10.1016/j.dsx.2019.06.011. Epub 2019 Jun 11. PMID: 31405646.	They evaluate symptoms of depression.
8	Rajabi-Naeeni M, Dolatian M, Qorbani M, Vaezi AA. Effect of omega-3 and vitamin D co-supplementation on psychological distress in reproductive-aged women with pre-diabetes and hypovitaminosis D: A randomized controlled trial. Brain Behav. 2021 Nov;11(11):e2342. doi: 10.1002/brb3.2342. Epub 2021 Sep 2. PMID: 34473420; PMCID: PMC8613419.	Psychological complications in women of childbearing age are evaluated.
9	Wenclewska S, Szymczak-Pajor I, Drzewoski J, Bunk M, Śliwińska A. Vitamin D Supplementation Reduces Both Oxidative DNA Damage and Insulin Resistance in the Elderly with Metabolic Disorders. Int J Mol Sci. 2019 Jun 13;20(12):2891. doi: 10.3390/ijms20122891. PMID: 31200560; PMCID: PMC6628266.	They evaluate DNA damage due to oxidation.
10	Miller EG, Nowson CA, Dunstan DW, Kerr DA, Menzies D, Daly RM. Effects of whey protein plus vitamin D supplementation combined with progressive resistance training on glycaemic control, body composition, muscle function and cardiometabolic risk factors in middle-aged and older overweight/obese adults with type 2 diabetes: A 24-week randomized controlled trial. Diabetes Obes Metab. 2021 Apr;23(4):938-949. doi: 10.1111/dom.14299. Epub 2021 Jan 19. PMID: 33369020.	They evaluate body composition and muscle function.
11	Chatterjee R, Fuss P, Vickery EM, LeBlanc ES, Sheehan PR, Lewis MR, Dolor RJ, Johnson KC, Kashyap SR, Nelson J, Pittas AG; D2d Research Group. Vitamin D Supplementation for Prevention of Cancer: The D2d Cancer Outcomes (D2dCA) Ancillary Study. J Clin Endocrinol Metab. 2021 Aug 18;106(9):2767-2778. doi: 10.1210/clinem/dgab153. PMID: 33693713; PMCID: PMC8372641.	Vitamin D and cancer prevention.
12	Ebrahimkhani S, Ghavamzadeh S, Mehdizadeh A. The effects of vitamin D and curcuminoids supplementation on anthropometric measurements and blood pressure in type 2 diabetic patients with coexisting hypovitaminosis D: A double-blind, placebo-controlled randomized clinical trial. Clin Nutr ESPEN. 2020 Jun;37:178-186. doi: 10.1016/j.clnesp.2020.02.017. Epub 2020 Mar 20. PMID: 32359741.	They measure anthropometric factors.
13	Imanparast F, Mashayekhi FJ, Kamankesh F, Rafiei F, Mohaghegh P, Alimoradian A. Improving the endothelial dysfunction in type 2 diabetes with chromium and vitamin D3 byreducing homocysteine and oxidative stress: A randomized placebo-controlled trial. J Trace Elem Med Biol. 2020 Dec;62:126639. doi: 10.1016/j.jtemb.2020.126639. Epub 2020 Aug 31. PMID: 32971450.	They measure endothelial dysfunction.
14	Asemi Z, Raygan F, Bahmani F, Rezavandi Z, Talari HR, Rafiee M, Poladchang S, Darooghegi Mofrad M, Taheri S, Mohammadi AA, Esmaillzadeh A. The effects of vitamin D, K and calcium co-supplementation on carotid intima-media thickness and metabolic status in overweight type 2 diabetic patients with CHD. Br J Nutr. 2016 Jul;116(2):286-93. doi: 10.1017/S0007114516001847. Epub 2016 May 20. PMID: 27198036.	They measure the thickness of the carotid artery.

15	Imanparast F, Javaheri J, Kamankesh F, Rafiei F, Salehi A, Mollaaliakbari Z, Rezaei F, Rahimi A, Abbasi E. The effects of chromium and vitamin D3 co-supplementation on insulin resistance and tumor necrosis factor-alpha in type 2 diabetes: a randomized placebo-controlled trial. Appl Physiol Nutr Metab. 2020 May;45(5):471-477. doi: 10.1139/apnm-2019-0113. Epub 2019 Oct 8. PMID: 31593637.	They evaluate tumor necrosis.

Table S4. Summary of Findings (SoF) and quality of evidence (GRADE) for vitamin D treatment in patients with diabetes mellitus.

Certainty Assessment							№ of Patients		Effect		Quality of Evidence (GRADE)	Importance
№ of Studies	Study Design	Risk of Bias	Inconsistency	Indirectness	Imprecision	Other Considerations	Vitamin D	Control Group	Relative (95% CI)	SMD (95% CI)		
**BMI**												
**2**	RCT	Not Serious	Serious	Not serious	Serious	None	94	108	-	−1.37 (−2.85 to 0.11)	⊕◯◯◯ Very low	CRITICAL
**Vitamin D**												
3	RCT	Not Serious	Very serious	Not serious	Serious	None	122	138	-	31.19 (28.27 to 34.11)	⊕◯◯◯ Very low	CRITICAL
HbA1c												
9	RCT	Not serious	Very serious	Not serious	Serious	None	349	354	-	−0.19(−0.31 to −0.07)	⊕⊕◯◯ Low	CRITICAL
**25-Hydroxyvitamin**												
3	RCT	Not Serious	Very serious	Not serious	Serious	None	108	104	-	19.80 (18.16 to 21.44)	⊕⊕◯◯ Low	IMPORTANT
**HOMA-IR**												
5	RCT	Not serious	Serious	Not serious	Serious	None	246	251	-	−1.36 (−1.38 to −1.14)	⊕⊕◯◯ Low	IMPORTANT
**Homa-β**												
2	RCT	Serious	Serious	Not serious	Serious	None	109	100	-	0.71 (0.63 to 0.80)	⊕◯◯◯ Very low	IMPORTANT

						**HDL cholesterol**						
8	RCT	Serious	Serious	Not serious	Serious	None	1092	1098	-	0.07 (0.05 to 0.09)	⊕◯◯◯ Very low	IMPORTANT
						**LDL cholesterol**						
8	RCT	Serious	Serious	Not serious	Serious	None	1092	1098	-	−0.40 (−0.45 to −0.34)	⊕⊕◯◯ Low	IMPORTANT
						**PTH**						
4	RCT	Serious	Serious	Not serious	Serious	None	165	157	-	−0.14(−2.14 to 1.86)	⊕◯◯◯ Very low	IMPORTANT
						**Calcium**						
3	RCT	Serious	Serious	Not serious	Serious	None	138	148	-	−0.01(−0.05 to 0.03)	⊕◯◯◯ Very low	IMPORTANT
						**Total cholesterol**						
9	RCT	Serious	Serious	Not serious	Serious	None	1123	1129	-	−0.46 (−0.52 to −0.40)	⊕⊕◯◯ Low	IMPORTANT
						**IL-6**						
3	RCT	Serious	Serious	Not serious	Serious	None	1292	1240	-	−0.36 (−0.81 to 0.10)	⊕◯◯◯ Very low	IMPORTANT
						**Phosphorus**						
2	RCT	Serious	Serious	Not serious	Serious	None	89	83	-	−0.12 (−0.30 to 0.05)	⊕◯◯◯ Very low	IMPORTANT
						**IL-1B**						
2	RCT	Serious	Serious	Not serious	Serious	None	1092	1098	-	0.001 (−0.001 to 0.001)	⊕⊕◯◯ Low	IMPORTANT
						**Fasting insulin**						

3	RCT	Serious	Serious	Not serious	Serious	None	162	164	-	−4.16 (−4.53 to −3.79)	⊕○○○ Very low	IMPORTANT
**Tryglecireds**												
8	RCT	Serious	Serious	Not serious	Serious	None	1092	1098	-	−0.52 (−0.55 to −0.50)	⊕○○○ Very low	IMPORTANT
**Protein C**												
4	RCT	Serious	Serious	Not serious	Serious	None	907	926	-	−0.31 (−0.40 to −0.21)	⊕⊕○○ Low	IMPORTANT
**Fasting Plasma/Blood Glucose**												
6	RCT	Serious	Serious	Not serious	Serious	None	265	268	-	−1.26 (−1.31 to −1.21)	⊕○○○ Very low	IMPORTANT
**SBP**												
5	RCT	Serious	Serious	Not serious	Serious	None	922	939	-	0.55 (−0.17 to 1.27)	⊕○○○ Very low	IMPORTANT
**DBP**												
5	RCT	Serious	Serious	Not serious	Serious	None	922	939	-	1.21 (0.65 to 1.77)	⊕⊕○○ Low	IMPORTANT
**QUIKI**												
5	RCT	Serious	Serious	Not serious	Serious	None	71	73	-	0.03(0.02 to 0.03)	⊕⊕○○ Low	IMPORTANT

SMD: Standard Mean Difference; **RCT:** Randomized clinical trial; **Quality of evidence:** High: The research provides a very good indication of the likely effect. The probability that the effect is different is low; Moderate: The research provides a good indication of the likely effect. The probability that the effect is substantially different is moderate; Low: The research gives some indication of the probable effect. However, the probability that the effect is substantially different is high; Very low: The research does not provide a reliable estimate of the probable effect. The probability that the effect is substantially different is very high. **Downgrading:** GRADE approach has four reasons for possible rate down the quality of evidence. Begins with the study designs (trials or observational studies), secondly downgrading the evidence

one level: (1) for study limitation if the majority of studies (>50%) was rated as high risk of bias; (2) for inconsistency, if heterogeneity was greater than the accepted low level (I^2 >40%); (3) for indirectness, directness was undoubled; (4) for imprecision, if meta-analysis had a small sample size (n <400) or confidence interval very wide.

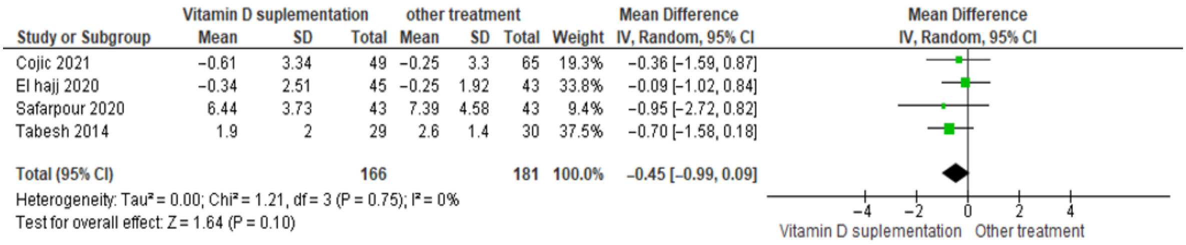

Figure S1. Forest plot Forest plot for outcome HOMA IR without author Huang et al., 2021.

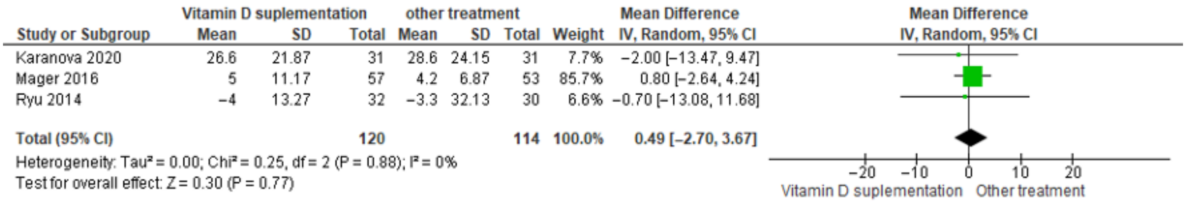

Figure S2. Forest plot for outcome PTH without author El Hajj et al., 2021.

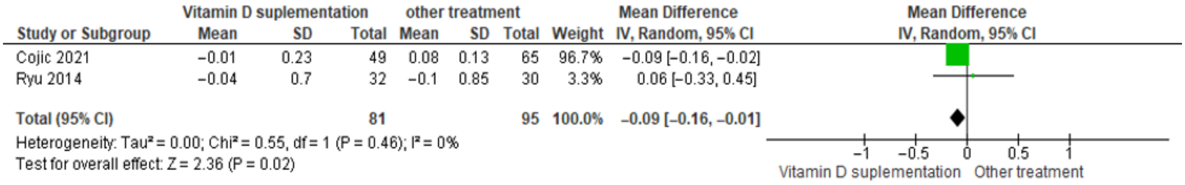

Figure S3. Forest plot for outcome Calcium, without author Mager et al., 2016.
